# Pure argyrophilic grain disease revisited: independent effects on limbic, neocortical, and striato-pallido-nigral degeneration and the development of dementia in a series with a low to moderate Braak stage

**DOI:** 10.1186/s40478-024-01828-6

**Published:** 2024-07-31

**Authors:** Osamu Yokota, Tomoko Miki, Hanae Nakashima-Yasuda, Hideki Ishizu, Takashi Haraguchi, Chikako Ikeda, Masato Hasegawa, Akinori Miyashita, Takeshi Ikeuchi, Naoto Nishikawa, Shintaro Takenoshita, Koichiro Sudo, Seishi Terada, Manabu Takaki

**Affiliations:** 1 Department of Neuropsychiatry, Okayama University Graduate School of Medicine, Dentistry and Pharmaceutical Sciences, 2-5-1 Shikata-cho, Okayama, 700-8558 Japan; 2 Okayama University Medical School, Okayama, Japan; 3 Department of Psychiatry, Kinoko Espoir Hospital, Okayama, Japan; 4 Department of Neuropathology, Pitié-Salpêtrière Hospital, AP-HP, Sorbonne University, Paris, France; 5 Institut du Cerveau - Paris Brain Institute - ICM, Inserm U1127, CNRS UMR7225, AP-HP, Pitié-Salpêtrière Hospital, Sorbonne University, Paris, France; 6 Department of Psychiatry, Zikei Hospital, Okayama, Japan; 7 Department of Neurology, National Hospital Organization Minami-Okayama Medical Center, Okayama, Japan; 8 Dementia Research Project, Tokyo Metropolitan Institute of Medical Science, Tokyo, Japan; 9 Department of Molecular Genetics, Brain Research Institute, Niigata University, Niigata, Japan; 10 Department of Neuropsychiatry, Okayama University Hospital, Okayama, Japan; 11 Department of Psychiatry, Tosa Hospital, Kochi, Japan; 12 Department of Neuropsychiatry, Okayama University Faculty of Medicine, Dentistry and Pharmaceutical Sciences, Okayama, Japan

**Keywords:** Argyrophilic grain, Globus pallidus, Hippocampal sclerosis, Striatum, Substantia nigra, Subthalamic nucleus

## Abstract

Agyrophilic grains (AGs) are age-related limbic-predominant lesions in which four-repeat tau is selectively accumulated. Because previous methodologically heterogeneous studies have demonstrated inconsistent findings on the relationship between AGs and dementia, whether AGs affect cognitive function remains unclear. To address this question, we first comprehensively evaluated the distribution and quantity of Gallyas-positive AGs and the severity of neuronal loss in the limbic, neocortical, and subcortical regions in 30 cases of pure argyrophilic grain disease (pAGD) in Braak stages I–IV and without other degenerative diseases, and 34 control cases that had only neurofibrillary tangles with Braak stages I–IV and no or minimal Aβ deposits. Then, we examined whether AGs have independent effects on neuronal loss and dementia by employing multivariate ordered logistic regression and binomial logistic regression. Of 30 pAGD cases, three were classified in diffuse form pAGD, which had evident neuronal loss not only in the limbic region but also in the neocortex and subcortical nuclei. In all 30 pAGD cases, neuronal loss developed first in the amygdala, followed by temporo-frontal cortex, hippocampal CA1, substantia nigra, and finally, the striatum and globus pallidus with the progression of Saito AG stage. In multivariate analyses of 30 pAGD and 34 control cases, the Saito AG stage affected neuronal loss in the amygdala, hippocampal CA1, temporo-frontal cortex, striatum, globus pallidus, and substantia nigra independent of the age, Braak stage, and limbic-predominant age-related TDP-43 encephalopathy (LATE-NC) stage. In multivariate analyses of 23 pAGD and 28 control cases that lacked two or more lacunae and/or one or more large infarctions, 100 or more AGs per × 400 visual field in the amygdala (OR 10.02, 95% CI 1.12–89.43) and hippocampal CA1 (OR 12.22, 95% CI 1.70–87.81), and the presence of AGs in the inferior temporal cortex (OR 8.18, 95% CI 1.03–65.13) affected dementia independent of age, moderate Braak stages (III–IV), and LATE-NC. Given these findings, the high density of limbic AGs and the increase of AGs in the inferior temporal gyrus may contribute to the occurrence of dementia through neuronal loss, at least in cases in a low to moderate Braak stage.

**Supplementary Information:**

The online version contains supplementary material available at 10.1186/s40478-024-01828-6.

## Introduction

Argyrophilic grains (AGs) are age-related lesions that are preferentially distributed in the limbic region and in which four-repeat tau is selectively accumulated. AGs usually develop in the 50’s or later, and the frequency of the lesions reaches around 10% in the 70’s [7]. The topographical progression of AGs in the central nervous system can be assessed using the staging system proposed by Saito et al. [53] (Saito AG stage). According to this staging system, AGs first develop in the ambient gyrus, hippocampal CA1, and amygdala (stage I), and subsequently, in the transentorhinal cortex, subiculum, lateral occipitotemporal gyrus, and anterior portion of the superior temporal gyrus (stage II). Further, AGs progress to the inferior temporal gyrus, insular cortex, cingulate gyrus, and gyrus rectus (stage III). With the progression of AGs, pretangles also occur in the basal ganglia and brain stem nuclei [18, 20, 21, 24, 25, 32, 34, 57]. Granular fuzzy astrocytes (GFAs), which were originally called bush-like astrocytes and are now classified as a subtype of age-related tau astrogliopathy (ARTAG), are consistently noted in limbic regions [5, 67]. The occurrence of GFAs may be an independent factor of the formation of AGs [26, 68], and AGs and ARTAG share tau filaments with identical cryo-electron microscopy structures [58]. Phosphorylation and conformation change of tau, fibril formation detected by the Gallyas method, and activation of autophagy in GFAs occur with the progression of the Saito AG stage [18, 37]. Some cases with Saito AG stage III have AGs in the extensive neocortical region and subcortical nuclei also, being called a diffuse form of argyrophilic grain disease (AGD) [35]. Due to severe tau pathologies, diffuse form AGD may be a subtype useful to understand the effects of AGs on neuronal loss in the central nervous system and related cognitive and motor dysfunction. However, reports of previous clinical and pathological findings in the diffuse form of AGD are very limited.

The effect of AGs on the function of the central nervous system that has been explored is mainly cognitive dysfunction. First, Braak et al. [6] examined 16 cases with dementia that lacked macroscopically detectable infarctions and were almost devoid of the neurofibrillary changes of Alzheimer’s disease, and they pointed out that a potential pathological base of dementia in eight cases was AGs. Thereafter, Saito et al. [53] reported that extensive distribution of AGs corresponding to Sato AG stage III was closely associated with dementia in cases without other lesions that can cause cognitive impairment. Subsequently, several researchers reported a potential relationship between AGs and cognitive function or psychiatric conditions: the significantly higher frequency of AGs in cases that clinically showed late-onset psychosis without dementia rather than normal control cases [39], the significantly higher frequency of AGs in corticobasal degeneration (CBD) cases with a cognitive-predominant presentation rather than CBD cases with corticobasal syndrome [54], and the significantly higher frequency of suicide in forensic autopsy cases with AGs rather than those without AGs [69]. On the other hand, however, there are also several studies that failed to reveal a relationship between AGs and cognitive impairment [17, 41, 48, 50]. Although these results are contradictory, because the procedures of histological evaluation of AGs including the staining method and the case selection criteria associated with the pathological background in subjects were quite varied in previous studies, whether AGs have some effect on cognitive function or not remains a matter of controversy.

The primary aims of this study were to clarify (1) whether AGs have an effect on the neuronal loss that is closely associated with cognitive impairment, and (2) whether the presence of AGs is an independent factor in the development of dementia. To address these issues, we pathologically examined 30 cases of pure AGD (pAGD) that had Gallyas-positive AGs but lacked other pathological changes that can result in neuronal loss and 34 control cases that had only neurofibrillary tangles (NFTs) in Braak stages I–IV and no or minimal Aβ deposits selected from our autopsy series. Of the 30 pAGD cases, three were classified as diffuse-form pAGD, and they had evident neuronal loss not only in the limbic region but also in the neocortex and subcortical nuclei. In all pAGD cases including diffuse-form cases, the severity of neuronal loss gradually increased not only in the limbic system but also in the temporo-frontal cortex, basal ganglia, and brain stem nuclei with the progression of Saito AG stage. In multivariate analyses of all pAGD and control cases, the progression of AGD had an effect on neuronal loss in the striatum, globus pallidus, substantia nigra, hippocampal CA1, temporo-frontal cortex, cingulate gyrus, and insular cortex independent of the age at death, Braak NFT stage, and LATE-NC. Further, 100 or more AGs per × 400 visual field in the amygdala, 100 or more AGs per × 400 visual field in the hippocampal CA1, and the presence of AGs in the inferior temporal cortex affected the occurrence of dementia independent of the age at death, Braak NFT stage, and LATE-NC.

## Materials and methods

### Subjects

Autopsies were carried out after informed consent was obtained from family members, and all experiments in this study were approved by the ethical committees of the Okayama University Graduate School of Medicine, Dentistry and Pharmaceutical Sciences, the National Hospital Organization Minami-Okayama Medical Center, Zikei Institute of Psychiatry, the Tokyo Metropolitan Institute of Medical Science, and Niigata University. From 1,125 autopsy cases who had died in psychiatric hospitals or neurological departments of general hospitals and were registered in the database at the Department of Neuropsychiatry, Okayama University Graduate School of Medicine, Dentistry and Pharmaceutical Sciences as of the end of December 2022, we first selected 475 cases for which the data on Saito AG stage were available. In our laboratory, AGs in the central nervous system were routinely evaluated on sections stained with Gallyas silver stain (Gallyas method), and the Saito AG stage was determined in all cases [6, 53]. Further, AGs were confirmed by being labeled with AT8 and an anti-4R tau antibody but not with an anti-3R tau antibody. From the database, other fundamental pathological data in all of these cases, i.e., Braak NFT stage [9], Thal Aβ phase [61], CERAD neuritic plaque score [38], pathological subtypes of Lewy body disease (LBD) [36, 63], Braak Parkinson’s disease (PD) stage [8], the semiquantitative scores of tufted astrocytes, astrocytic plaques, and GFAs [37, 67], the stage of limbic-predominant age-related TDP-43 encephalopathy pathologic change (LATE-NC) [43, 44], histological subtypes of frontotemporal lobar degeneration with TDP-43-positive inclusions [11, 27, 30], the presence or absence of fused in sarcoma (FUS) pathology [29], the presence or absence of *C9ORF72* mutation-related p62-positive inclusions in the cerebellar granular cell layer [31, 47], the semiquantitative data regarding the severity of neuronal loss with gliosis in representative anatomical regions in the neocortex, basal ganglia, brain stem nuclei, cerebellum, and spinal cord (defined later), and the data of vascular lesions were extracted. The pathological assessments of all cases were routinely carried out by a senior neuropathological researcher (OY) with or without another neuropathological researcher (TM, HI, or ST).

Among 475 cases, 77 cases had AGs. To minimize the influence of various pathological conditions except for AGs on the analysis of neuronal loss, we excluded 47 AGD cases that had at least one of the following pathologies: NFTs with Braak stages V–VI, Lewy body disease (LBD), tufted astrocytes, astrocytic plaques, frontotemporal lobar degeneration with TDP-43-positive inclusions (FTLD-TDP) [11, 27, 30], amyotrophic lateral sclerosis with TDP-43-positive inclusions (ALS-TDP) [11], FTLD or ALS with FUS-positive pathologies [11], globular glial tauopathy [2], and other various established neurodegenerative diseases (e.g., multiple system atrophy, spinocerebellar degeneration, myotonic dystrophy, Huntington’s disease, dentatorubral–pallidoluysian atrophy, post-encephalitic parkinsonism, Bechet’s disease, leukodystrophies, Alexander disease, neuronal ceroid lipofuscinoses, and Marchiafava–Bignami disease). Cases having findings of global ischemia were also excluded, but cases bearing LATE-NC were not. Finally, 30 cases having AGs were extracted, and we regarded these cases as pAGD (Table 1).

**Table 1:** Demographic data in pAGD and control cases

	pAGD					Control (n = 34)
	All pAGD (n = 30)	Diffuse form (n = 3)	Saito AG stage III (n = 3)	Saito AG stage II (n = 12)	Saito AG stage I (n = 12)	
Female (%)	43.3	33.3	66.7	58.3	25.0	36.4
Age at death (mean ± SD)	76.5 ± 9.0	76.3 ± 14.6	85.7 ± 4.2	77.7 ± 9.1	73.1 ± 7.2	65.3 ± 8.8
Brain weight (g, mean ± SD)	1190.5 ± 154.8	1114.0 ± 104.7	1107.7 ± 208.5	1191.6 ± 127.8	1222.9 ± 177.9	1210.3 ± 174.7
Braak NFT stage (n, %)						
Stage 6	0 (0.0)	0 (0.0)	0 (0.0)	0 (0.0)	0 (0.0)	0 (0.0)
Stage 5	0 (0.0)	0 (0.0)	0 (0.0)	0 (0.0)	0 (0.0)	0 (0.0)
Stage 4	7 (0.0)	1 (33.3)	1 (33.3)	4 (33.3)	1 (8.3)	2 (5.9)
Stage 3	3 (0.0)	0 (0.0)	1 (33.3)	0 (0.0)	2 (16.7)	4 (11.8)
Stage 2	17 (23.3)	1 (33.3)	1 (33.3)	7 (58.3)	8 (66.7)	10 (29.4)
Stage 1	3 (10.0)	1 (33.3)	0 (0.0)	1 (8.3)	1 (8.3)	18 (52.9)
Stage 0	0 (0.0)	0 (0.0)	0 (0.0)	0 (0.0)	0 (0.0)	0 (0.0)
Median	2.5	2.0	3.0	2.0	2.0	1.0
25th percentile	1.0	1.0	2.0	2.0	2.0	1.0
75th percentile	2.0	4.0	4.0	4.0	3.0	2.0
Thal phase (n, %)						
Phase 5	0 (0.0)	0 (0.0)	0 (0.0)	0 (0.0)	0 (0.0)	0 (0.0)
Phase 4	1 (3.3)	0 (0.0)	1 (33.3)	0 (0.0)	0 (0.0)	0 (0.0)
Phase 3	4 (13.3)	0 (0.0)	1 (33.3)	3 (25.0)	0 (0.0)	0 (0.0)
Phase 2	5 (16.7)	0 (0.0)	0 (0.0)	2 (16.7)	3 (25.0)	2 (5.9)
Phase 1	4 (13.3)	1 (33.3)	0 (0.0)	2 (16.7)	1 (8.3)	9 (26.5)
Phase 0	16 (53.3)	2 (66.7)	1 (33.3)	5 (41.7)	8 (66.7)	23 (67.6)
Median	0.0	0.0	3.0	1.0	0.0	0.0
25th percentile	0.0	0.0	0.0	0.0	0.0	0.0
75th percentile	2.0	1.0	4.0	2.8	1.8	1.0
Lewy body disease (n, %)	0 (0.0)	0 (0.0)	0 (0.0)	0 (0.0)	0 (0.0)	0 (0.0)
LATE stage (n, %)						
Stage 3	0 (0.0)	0 (0.0)	0 (0.0)	0 (0.0)	0 (0.0)	0 (0.0)
Stage 2	6 (20.0)	2 (66.7)	1 (33.3)	3 (25.0)	0 (0.0)	0 (0.0)
Stage 1	2 (6.7)	0 (0.0	0 (0.0)	1 (8.3)	1 (8.3)	0 (0.0)
Stage 0	22 (73.3)	1 (33.3)	2 (66.7)	8 (66.7)	11 (91.7)	34 (100.0)
Median	0.0	2.0	0.0	0.0	0.0	0.0
25th percentile	0.0	1.0	0.0	0.0	0.0	0.0
75th percentile	0.75	2.0	1.0	1.25	0.0	0.0
Dementia, n (%)^a^	12/23 (52.2)	3/3 (100.0)	2/2 (100.0)	4/9 (44.4)	3/9 (33.3)	2/28 (7.1)

*pAGD* pure argyrophilic grain disease, *diffuse form* diffuse form of pAGD. All diffuse form cases fit the criteria of Saito AG stage III. *n* the number of cases, *SD* standard deviation, *NFT* neurofibrillary tangle, *LATE* limbic-predominant age-related TDP-43 encephalopathy. *n.e.* not examined.

^a^The number of cases with dementia but without vascular lesions and the number of all cases without vascular lesions in each group are indicated. Cases having one or more large infarction or two or more lacunae in the cortical and/or subcortical regions were excluded

Then, we also extracted 34 control cases that had NFTs in Braak stages I to IV with no or mild Aβ deposits with Thal phases 1 or 2 from our autopsy series, corresponding to the definition of primary age-related tauopathy [12]. Cases bearing AGs or other neurological diseases noted above were not included in this group. In these 34 control cases, no case had LATE-NC (Table 1).

The data regarding dementia in all pAGD and control cases were extracted from our data base. The presence of dementia was determined based on clinical diagnosis, cognitive impairment with the necessity of support in instrumental activity of daily living that was noted in the clinical summary, and/or the retrospective interview of physicians who had provided long-term treatment at least in the late stage of the clinical course [3]. These clinical data were known before the pathological examinations of each case. The data regarding dementia were available in all 30 pAGD cases and 33 of 34 control cases.

### Neuropathological examination

Brain tissue samples were fixed post mortem with 10% formaldehyde and embedded in paraffin. Ten-μm-thick sections from the frontal, temporal, parietal, occipital, insular, and cingulate cortices, hippocampus, amygdala, basal ganglia, midbrain, pons, medulla oblongata, and cerebellum were prepared. These sections were stained with hematoxylin–eosin (H&E), Klüver–Barrera (KB), the Gallyas method, and modified Bielschowsky silver methods.

Paraffin sections were immunostained by the immunoperoxidase method using 3, 3′-diaminobenzidine tetrahydrochloride. Six-μm-thick paraffin sections were immunostained by the immunoperoxidase method using 3,3′-diaminobenzidine tetrahydrochloride. Deparaffinized sections were incubated with 1% H_2_O_2_ in methanol for 20 min to eliminate endogenous peroxidase activity in the tissue. Sections were treated with 0.2% TritonX-100 for 5 min and washed in phosphate-buffered saline (PBS, pH 7.4). After blocking with 10% normal serum, sections were incubated overnight at 4 °C with one of the primary antibodies (Supplementary Table 1) in 0.05 M Tris–HCl buffer, pH 7.2, containing 0.1% Tween and 15 mM NaN_3_. After three 10-min washes in PBS, sections were incubated in biotinylated anti-rabbit, -mouse, or -pig secondary antibody for 1 h, and then in avidin-biotinylated horseradish peroxidase complex (ABC Elite kit, Vector, Newark, CA, USA) for 1 h. The peroxidase labeling was visualized with diaminobenzidine as the chromogen.

### Semiquantitative assessment of the quantity of AGs

AGs were semi-quantitatively assessed in the representative anatomical regions in all pAGD and control cases. Regions examined were the ambient gyrus, amygdala, hippocampal CA1, transentorhinal cortex, subiculum, anterior portion of the superior temporal gyrus, lateral occipitotemporal gyrus, insular cortex, inferior temporal gyrus, middle frontal gyrus, primary motor cortex, inferior parietal lobule, peristriate region, striate region, caudate nucleus, putamen, globus pallidus, periaqueductal gray, substantia nigra, pontine tegmentum, medullary tegmentum, and spinal anterior horns (Table 2). Sections stained with the Gallyas method were employed. The grading system of the quantity of AGs used in this study was a modified version of that used in an original report of Saito AG stage [53] (i.e., one to 19 AGs per × 400 visual field was defined as ± .): −, no grain; ± , more than one to 19 AGs per × 400 visual field; + , 20 to 50 AGs per × 400 visual field; +  + , 51 to 100 AGs per × 400 visual field; +  +  + , more than 100 AGs per × 400 visual field. Control cases had no AG in any region.

**Table 2:**
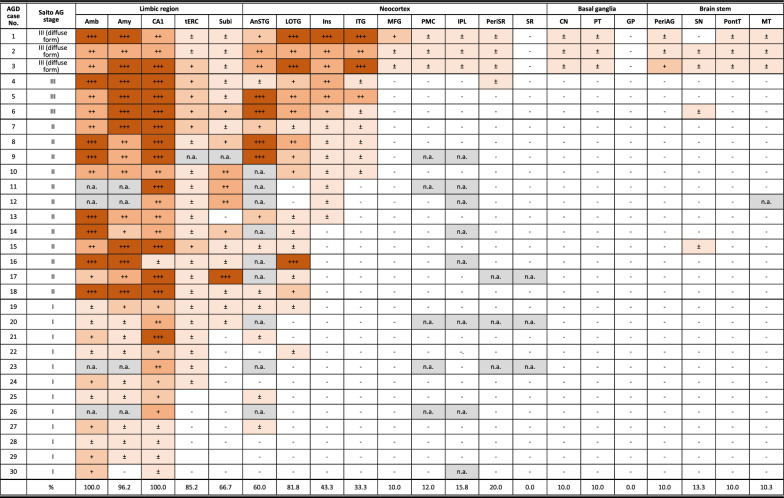
Distribution of AGs in pAGD cases

*Amb* ambient gyrus, *Amy* amygdala, *tERC* transentorhinal cortex, *Subi* subiculum, *LOTG* lateral occipitoteporal gyrus, *ITG* inferior temporal gyrus, *Ins* insular cortex, *AnSTG* anterior portion of the superior temporal gyrus, *MFG* middle frontal gyrus, *PMC* primary motor cortex, *IPL* inferior parietal lobule, *PeriSR* peristriate region, *SR* striate region (primary visual cortex), *CN* caudate nucleus, *PT* putamen, *GP* globus pallidus, *PeriAG* periaqueductual gray, *SN* substantia nigra, *PontT* pontine tegmentum, *MT* medullary tegmentum, *AH* spinal anterior horn

−: no argyrophilic grain, ± : less than 20 AGs per × 400 visual field, + : 20 to 50 AGs per × 400 visual field, +  + : 50 to 100 AGs per × 400 visual field, +  +  + : 100 or more AGs per × 400 visual field. n.a.: not available

### Staging of the distribution of AGs (modified Saito staging system)

The distribution of AGs was assessed on sections stained with the Gallyas method using the Saito AG stage in all pAGD cases [53]. In the original description of the Saito AG stage, the handling of anatomical regions having a small number of AGs (i.e., one to 19 AGs per × 400 visual field) in the determination of the Saito stage was not described [53]. Therefore, in this study, we operationally classified pAGD cases into each Saito AG stage:

Saito AG stage 0: there was no argyrophilic grain in any region in the cerebrum and brain stem.

Saito AG stage I: one to 50 AGs per × 400 visual field were present in the limbic region (i.e., the ambient gyrus, amygdala, and/or the anterior portion of hippocampal CA1). There were fewer than 20 AGs per × 400 visual field in the superior temporal gyrus at the level of the temporal tip, lateral occipitotemporal gyrus, transentorhinal cortex, and subiculum.

Saito AG stage II: there were AGs that fit stage I, and 20 or more AGs per × 400 visual field are additionally present in at least one of the following four regions: (i) the superior temporal gyrus at the level of the temporal tip, (ii) the lateral occipitotemporal gyrus at the level of the amygdala or hippocampus, (iii) the transentorhinal cortex, and (iv) the subiculum. There were no or fewer than 20 AGs in the insular cortex.

Saito AG stage III: there were AGs that fit stage II, and 20 or more AGs per × 400 visual field were additionally present in the insular cortex.

The diffuse form of pAGD was defined as one or more AGs in the limbic system, temporal cortex, and all of the following regions: (i) all of the middle frontal gyrus, motor cortex, inferior parietal lobule, and occipital cortex, (ii) the striatum (the caudate nucleus and/or putamen), (iii) the midbrain (the periaqueductal gray and/or substantia nigra), (iv) the pontine tegmentum, and (v) the tegmentum in the medulla oblongata. As a result, all of our diffuse form pAGD cases also fit the definition of Saito AG stage III.

### Semiquantitative assessment of neuronal loss with glial proliferation

In all pAGD and control cases, the severity of neuronal loss with gliosis in the cerebral cortex was assessed on H&E- and KB-stained sections according to the four-point staging system employed in our previous studies (Supplementary Fig. 1) [64, 65]: stage 0, neither neuronal loss not gliosis was observed; stage 1, slight neuronal loss and gliosis were observed only in the superficial layers; stage 2, obvious neuronal loss and gliosis were found in cortical layers II and III, and status spongiosis and relative preservation of neurons in layers V and VI were often present; and stage 3, pronounced neuronal loss with gliosis was found in all cortical layers, and adjacent subcortical white matter exhibits prominent fibrous gliosis.

In the basal ganglia and brainstem nuclei, the degree of neuronal loss with gliosis was assessed on H&E- and KB-stained sections according to the four-point staging system employed in our previous studies [64, 65] (Supplementary Fig. 2): stage 0, neither neuronal loss nor gliosis was observed; stage 1, mild gliosis and mild neuronal loss were present; stage 2, neuronal loss and gliosis were moderate, but tissue rarefaction was absent; and stage 3, severe neuronal loss, severe fibrous gliosis, and tissue rarefaction were observed.

The severities of the degeneration of the corticospinal tract at the level of the cerebral peduncle and the medulla oblongata and that of the frontopontine tract at the level of the cerebral peduncle were assessed as follows: stage 0, neither loss of myelin nor glial proliferation; stage 1, slight myelin loss and gliosis without atrophy of the tract; stage 2, evident myelin loss and gliosis with slight atrophy of the tract; stage 3, evident myelin loss and gliosis with severe atrophy of the tract.

### Semiquantitative assessment of NFTs and GFAs

NFTs in the representative anatomical regions were assessed according to a four-point staging system in cases of diffuse form pAGD: stage 0, no lesion in the anatomical region; stage 1, more than one lesion in the anatomical region but less than one lesion per × 200 visual field; stage 2, one to ten lesions per × 200 visual field; stage 3, 11 to 20 per × 200 visual field; stage 4, 21 to 30 per × 200 visual field; and stage 5, 31 or more per × 200 visual field.

GFAs in the middle frontal cortex, caudate nucleus, putamen, and amygdala were semiquantitatively assessed according to a three-point staging system (GFA stage) in cases of diffuse from of pAGD: stage 0, no lesion in the anatomical region; stage 1, one or more lesions in regions examined but less than one lesion per × 200 visual field; and stage 2, one or more lesions per × 200 visual field.

### Tau immunoblotting

Frozen brain tissue from the hippocampus, amygdala, inferior temporal gyrus, middle frontal gyrus, and caudate nucleus of the right hemisphere in two diffuse-form pAGD cases (Cases 1 and 2 in Supplementary Table 2 and Supplementary file 1) was available. These samples were used for Western blotting according to methods described previously [59]. Brain samples (0.5 g) from patients were individually homogenized in 20 ml of homogenization buffer (HB: 10 mM Tris–HCl, pH 7.5, containing 0.8 M NaCl, 1 mM EGTA, and 10% sucrose). Sarkosyl was added to the lysates (final concentration: 2%), which were then incubated for 30 min at 37 °C and centrifuged at 27,000 *g* for 10 min at 25 °C. The supernatant was divided into tubes (each 1.3 ml) and centrifuged at 166,000 *g* for 20 min at 25 °C. The pellets were further washed with 0.1% sarkosyl in a homogenization buffer (0.5 ml/tube) and centrifuged at 166,000 *g* for 20 min. The resulting pellets were used as the sarkosyl-insoluble fraction (ppt). The sarkosyl-ppt was sonicated in 50 μl (/tube) of 30 mM Tris–HCl (pH 7.5) and solubilized in 2 × sample buffer. Samples were run on gradient 4–20% polyacrylamide gels and electrophoretically transferred to PVDF membranes. Residual protein-binding sites were blocked by incubation with 3% gelatin (Wako) for 10 min at 37 °C, followed by overnight incubation at room temperature with primary anti-tau antibodies (AT8, mouse, monoclonal, 1:500; T46, mouse, monoclonal, 1:1,000). The membrane was incubated for 1 h at room temperature with anti-mouse IgG (BA-2000, Vector Lab) or anti-rabbit IgG (BA-2000, Vector Lab), then incubated for 30 min with avidin-horseradish peroxidase (Vector Lab), and the reaction product was visualized by using 0.1% DAB and 0.2 mg/ml NiCl_2_ as the chromogen.

### Genetic analysis of MAPT mutation and ApoE genotype

Genomic DNA extracted from autopsy brains (middle frontal gyrus) was used to determine pathological variants of MAPT and APOE genotypes in two diffuse form pAGD cases (cases 1 and 2 in Supplementary Table 2 and Supplementary file 1). Primer sequences and PCR conditions are available upon request. The concentration of extracted genomic DNA was measured using NanoDrop OneC (Thermo Fisher Scientific, Waltham, MA, USA), and its quality control was also performed by an Agilent 4200 TapeStation (Agilent Technologies, Santa Clara, CA, USA). For Sanger sequencing, the BigDye Terminator v3.1 Cycle Sequencing Kit (Thermo Fisher Scientific) was used, and the series of reactions was conducted according to the instruction manual.

### Immunoelectron microscopy

Sarkosyl-insoluble fractions extracted from two brains with diffuse form pAGD (cases 1 and 2 in Supplementary Table 2 and Supplementary file 1) were dropped onto carbon-coated nickel grids (Nissin EM, Tokyo, Japan). The grids were immunostained with an anti-phosphorylated tau monoclonal antibody (AT8, mouse, 1:200) and a secondary antibody conjugated to 5 nm gold particles (BBI Solutions, 1:50) as described [14]. Electron micrograph images were recorded with a JEOL JEM-1400 electron microscope (JEOL).

### Statistical analysis

Fisher’s exact test was used to compare the variables between two groups. Correlations between variables in pAGD cases were assessed by Spearman’s rank-order correlation test.

To assess the effects of predictor variables on the severity of neuronal loss in representative regions (amygdala, entorhinal cortex, hippocampal CA1, lateral occipitotemporal gyrus, inferior, middle, and superior temporal gyri, insular cortex, cingulate gyrus, middle frontal gyrus, orbital gyrus, and substantia nigra, respectively) in a combined group of 30 pAGD and 34 control cases, we performed multivariate ordered logistic regression analyses with neuronal loss stage (stages 0–3) as the dependent variable and the age at death, Braak NFT stage, Saito AG stage (stage 0, stage I, stage II, and stage III including diffuse form), and LATE-NC stage as independent variables. In the substantia nigra, the data of neuronal loss stages 1 and 2 were combined (stage 0, stages 1–2, and stage 3). We additionally performed multivariate ordered logistic regression analyses with neuronal loss stages in the amygdala and middle frontal gyrus as dependent variables and the age at death, Braak NFT stage, Saito AG stage (stage 0, stage I, stage II, and stage III including the diffuse form), LATE-NC stage, and GFA stage as independent variables. A Brant test was performed to check the proportional odds assumption, which was satisfied (*p* value ≥ 0.05).

Then, to assess the effects of predictor variables on the occurrence of neuronal loss in the caudate nucleus, putamen, and globus pallidus in the combined group of 30 pAGD and 34 control cases, we performed binomial logistic regression analyses with the presence of neuronal loss (neuronal loss stage 0/stages 1–3) as the dependent variable and the age at death, moderate Braak NFT stage (stages 0–II/stages III–IV), severe AGD (Saito AG stages 0–II/Saito AG stage III including the diffuse form), and moderate LATE-NC (stages 0–1/stage 2. No pAGD or control case had LATE-NC in stage 3) as independent variables. We additionally performed binomial logistic regression analyses with neuronal loss in the caudate nucleus and putamen as dependent variables and the age at death, moderate Braak NFT stage (stages 0–II/stages III–IV), severe AGD (Saito AG stages 0–II/Saito AG stage III including the diffuse form), moderate LATE-NC (stages 0–1/stages 2. No pAGD or control case had LATE-NC in stage 3), and the presence of GFA (GFA stage 0/GFA stages 1–2) as independent variables.

We examined the impacts of various pathological factors on the development of dementia in a combined group of pAGD and control cases using univariate binomial logistic regression analysis. To minimize the effect of vascular lesions on the development of dementia, cases that had one or more large infarctions and/or two or more lacunae in the neocortex or subcortical nuclei were excluded. As a result, 23 pAGD and 28 control cases were included in this analysis. Independent variables were the age at death, moderate Braak NFT stages (stages 0–II/stages III–IV), Aβ deposits (Thal phase 0/Thal phases 1–5), Saito AG stage (stages I, stage II, and stages II and III, respectively), three density ranges of AGs in the amygdala (one to 49, 50 to 99, and 100 or more per × 400 visual field, respectively), AGs in the amygdala (presence or absence), three density ranges of AGs in the hippocampal CA1 (one to 49, 50 to 99, or 100 or more per × 400 visual field, respectively), AGs in the hippocampal CA1 (presence or absence), AGs in the lateral occipitotemporal gyrus (presence or absence), AGs in the inferior temporal gyrus (presence or absence), AGs in the insular cortex (presence or absence), LATE-NC (presence or absence), moderate LATE-NC stage (stage 2, no pAGD or control case had LATE-NC in stage 3), and moderate to severe neuronal loss stage in the amygdala (stages 0–1/stages 2–3).

Then, to examine the independent effect of AGs on the development of dementia in a combined group of 23 pAGD and 28 control cases, we performed multivariate binomial logistic regression analyses with the age at death, Braak NFT stages III–IV (presence or absence), one of the pathological variables regarding AGs, and LATE-NC (presence or absence) as independent variables. A pathological variable regarding AGs submitted into each model as the independent variable was three density ranges of AGs in the amygdala (one to 49 AGs, 50 to 99 AGs, or 100 or more AGs per × 400 visual field, respectively), three density ranges of AGs in the hippocampal CA1 (one to 49 AGs, 20 to 99 AGs, or 100 or more AGs per × 400 visual field, respectively), AGs in the lateral occipitotemporal gyrus (presence or absence), or AGs in the inferior temporal gyrus (presence or absence), respectively.

Odds ratios (ORs) and 95% confidence intervals (CIs) were calculated. A *p* value < 0.05 was accepted as significant. Statistical analysis was performed using BellCurve for Excel 2.15 (Social Survey Research Information Co., Ltd., Tokyo, Japan) and EZR (Saitama Medical Center, Jichi Medical University, Saitama, Japan), which is a graphical user interface for R (The R Foundation for Statistical Computing, Vienna, Austria).

## Results

The demographic data of pAGD (n = 30) and control (n = 34) cases are shown in Table 1. The age at death of pAGD cases was significantly older than that of control cases (76.5 ± 9.0 years vs. 65.9 ± 9.4 years, *p* < 0.01, Mann–Whitney U test). The sex ratio did not differ between AGD and control groups (Fisher’s exact test). Among extracted 30 pAGD cases, six cases (20.0%) were classified as Saito AG stage III. Three of these six cases fit our pathological definition of diffuse form pAGD. Twelve pAGD cases (40.0%) were classified in Saito AG stage II, and 12 AGD cases (40.0%) were in Saito AG stage I.

### Clinical, pathological, and biochemical features in diffuse form pAGD cases

**Fig. 1:**
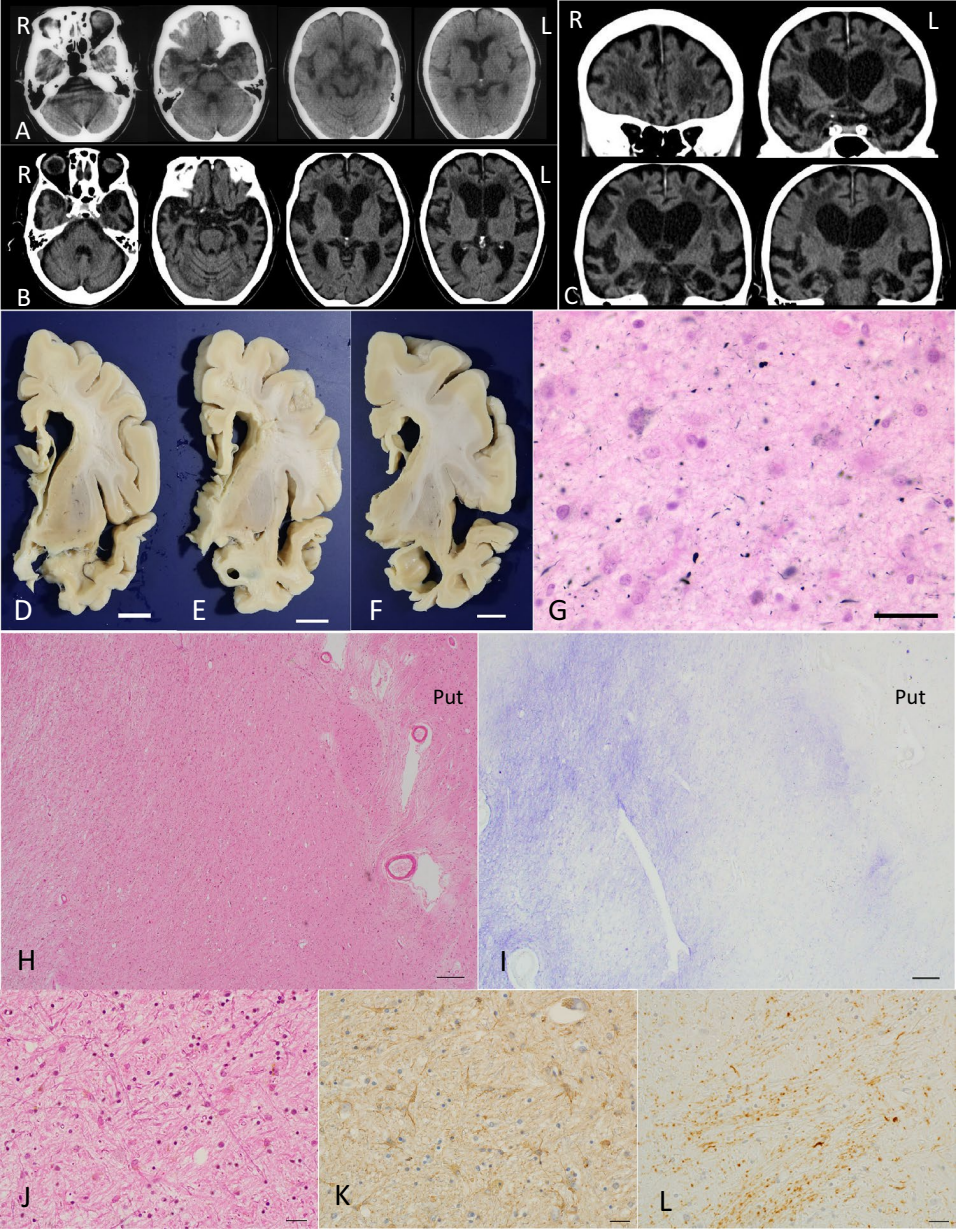
Radiological and pathological findings in case 1. **A** Axial CT images at the age of 74 years. Mild atrophy was noted in the amygdala, but the neocortex was almost completely spared. Bilateral Sylvian fissures were slightly dilated. **B**, **C** Axial (**B**) and coronal (**C**) CT images at age 86. Atrophy in the amygdala became severe. Atrophy in the neocortex was diffuse and symmetric but was slightly accentuated in the frontotemporal lobes. **D**–**F** Macroscopic findings of coronal sections of the left hemisphere. Scale bars = 1 cm. **G** AGs in the amygdala. Gallyas method. Scale bar = 30 μm. **H** Severe neuronal loss with gliosis in the globus pallidus. The degeneration is more evident in the internal segment (especially in the dorsal portion) than in the external segment in the site. Put: the putamen. H&E stain. Scale bar = 200 μm. **I** The same region shown in (**H**). Fibrillary gliosis is more evident in the internal segment rather than the external segment in the globus pallidus. Holzer stain. Scale bar = 200 μm. **J**–**L** Severe neuronal loss with gliosis in the globus pallidus revealed by H&E stain (**J**) and GFAP immunohistochemistry (**K**). Tau accumulation in the same region (**L**). AT8 immunohistochemistry. All scale bars = 20 μm. **A**–**C** Reprinted with permission from reference [66]

Clinical and genetic data and detailed clinical courses in diffuse form pAGD cases are described in Supplementary file 1 and summarized in Supplementary Table 1. A part of the clinical and pathological features in case 1 was briefly reported in Japanese [66]. The age at onset in the three cases ranged from 44 to 64 years. The disease duration ranged from 16 to 38 years. The initial symptoms were disinhibited behaviors and impairment of face recognition. Asymmetric rigidity was noted in two cases in the middle to late stage of the clinical course. Two cases for which frozen brain tissue was available lacked the MAPT mutation. Magnetic resonance imaging and computed tomography in cases 1 and 2 during the course revealed progressive atrophy in the limbic region, as well as in the neocortex and basal ganglia (Figs. 1A–C and 3A–D). Single-photon emission computed tomography (99mTc-ECD SPECT) [33] in the middle stage of the clinical course demonstrated the hypoperfusion in the limbic region in case 2 (Fig. 3E, F).

**Fig. 2:**
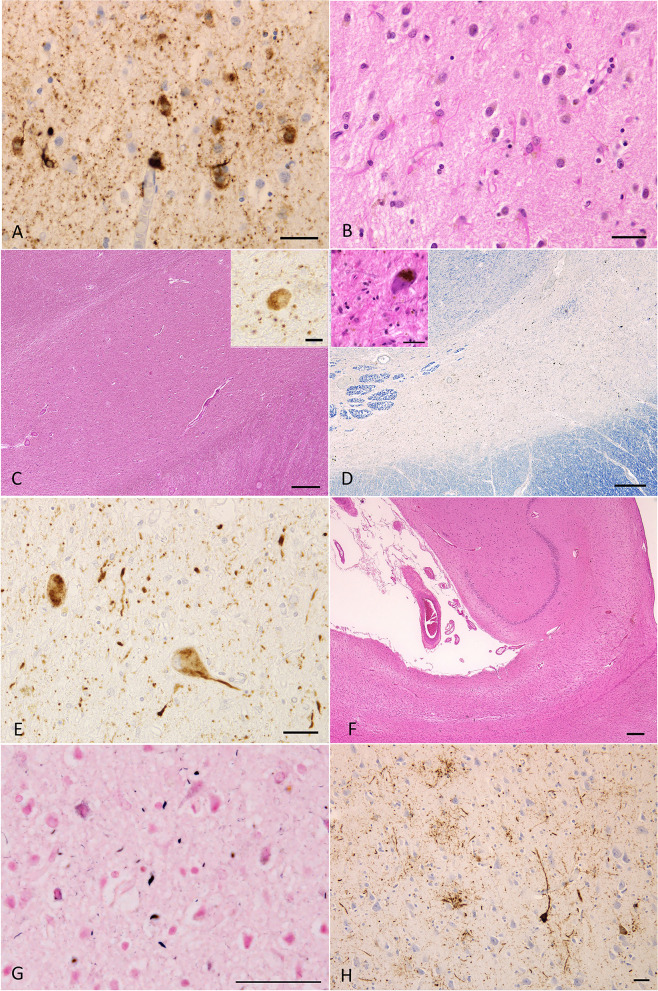
Pathological findings in case 1. **A** Tau-positive neurons, AGs, and granular dot-like lesions in neuropil in the caudate nucleus. AT8 immunohistochemistry. Scale bar = 30 μm. **B** Neuronal loss with astrocytosis in the caudate nucleus. H&E stain. Scale bar = 30 μm. **C** Macroscopically the volume of the subthalamic nucleus was spared, and neuronal loss was not noted. However, tau-positive neurons were found (inset). H&E stain. Scale bar = 300 μm (inset: AT8 immunohistochemistry, 10 μm). **D** Severe neuronal loss with gliosis (inset) in the substantia nigra. KB stain. Scale bar = 300 μm (inset: H&E stain, 30 μm). **E** AT8-immunopositive neurons, threads, and AGs in the substantia nigra. Scale bar = 30 μm. **F** Hippocampal sclerosis. H&E stain. Scale bar = 300 μm. **G** AGs in the middle frontal gyrus. Gallyas method. Scale bar = 50 μm. **H** Numerous GFAs in the superior frontal gyrus. AT8 immunohistochemistry. Scale bar = 30 μm

**Fig. 3:**
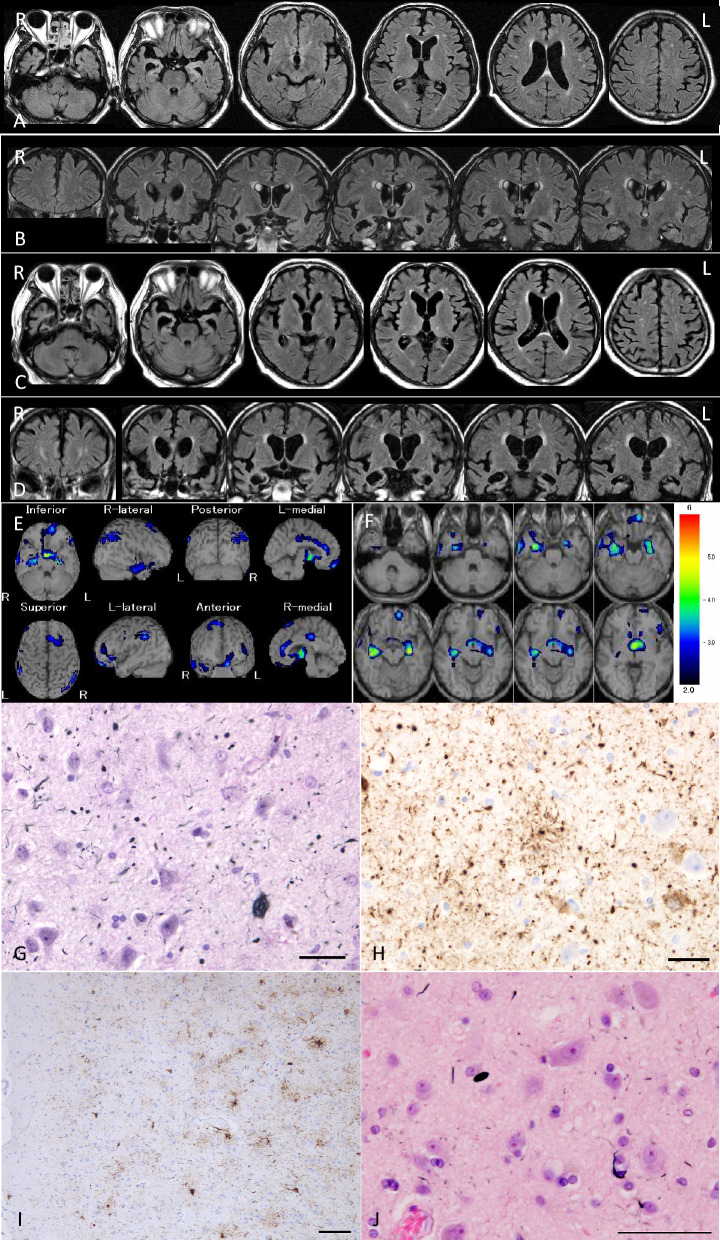
Radiological and pathological findings in case 2. **A**, **B** Axial (**A**) and coronal (**B**) MR images at the age of 69 years. The amygdala and adjacent parahippocampal gyrus show right side predominant atrophy. Temporal lobes are also atrophic, but frontal, parietal, and occipital cortices appear to be not involved. **C**, **D** Axial (**C**) and coronal (**D**) MR images at age 74. Right side-predominant atrophy in the amygdala and hippocampus become more evident. Mild frontal atrophy was also noted. **E**, **F** ^99m^Tc ECD brain perfusion single photon computed tomography (SPECT). SPECT Z-score maps compared with the standard database (the easy Z-score imaging system (eZIS) analysis software [33]). Blue, green, and yellow colors indicate a Z-score higher than 2.0. The amygdala rather than hippocampus exhibited hypoperfusion. **G** AGs in the amygdala. Gallyas method. Scale bar = 30 μm. **H** AT8-positive GFAs and AGs in the amygdala. Scale bar = 30 μm. **I** Numerous AT8-positive GFAs and some pretangles in the middle frontal gyrus. Scale bar = 100 μm. **J** In the same region of (**I**), Gallyas method demonstrated AGs and a coiled body. Scale bar = 50 μm

**Fig. 4:**
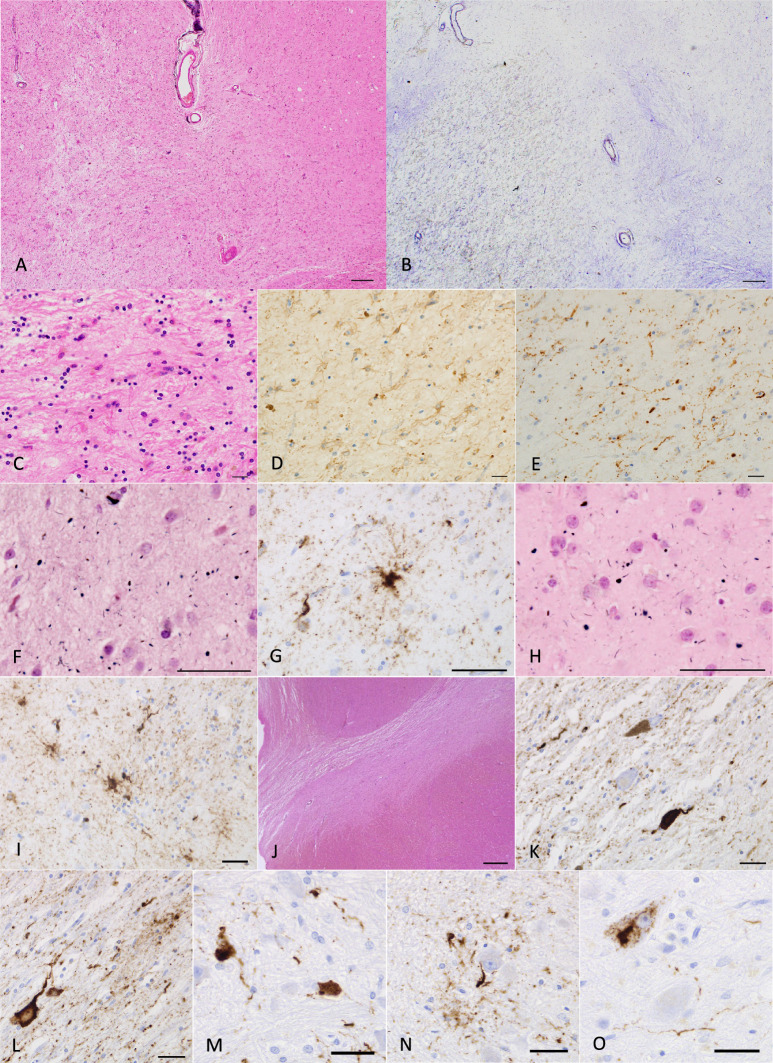
Pathological findings in case 2. **A** Severe neuronal loss with gliosis in the globus pallidus. The degeneration is more evident in the internal segment (left side) rather than external segment (right side) in the site. Especially, tissue rarefaction is severe in the dorsal portion of the internal segment. H&E stain. Scale bar = 200 μm. **B** Fibrillary gliosis is more evident in the internal segment than in the external segment in the globus pallidus. Holzer stain. Scale bar = 200 μm. **C**, **D** Severe gliosis in the globus pallidus demonstrated by H&E stain and GFAP immunohistochemistry. Scale bars = 20 μm. **E** AT8-positive threads and dot-like lesions in the globus pallidus. Scale bar = 20 μm. **F** AGs in the caudate nucleus. Gallyas method. Scale bar = 50 μm. **G** AT8-positive GFAs and a coiled body in the caudate nucleus. Scale bar = 50 μm. **H** Argyrophilic grains in the putamen. Gallyas method. Scale bar = 50 μm. **I** AT8-positive GFAs in the putamen. Scale bar = 50 μm. **J** Severe neuronal loss with gliosis in the substantia nigra. H&E stain. Scale bar = 600 μm. **K** AT8-positive NFT, threads, and AGs in the substantia nigra. Scale bar = 30 μm. **L** AT8-positive NFT, GFA, and threads in the subthalamic nucleus. Scale bar = 30 μm. **M** AT8-positive NFTs and threads in the pontine nucleus. Scale bar = 30 μm. **N** AT8-positive GFA and coiled body in the inferior olivary nucleus. Scale bar = 30 μm. **O** AT8-positive NFT and threads in the dentate nucleus in the cerebellum. Scale bar = 30 μm

Pathological findings in three diffuse form pAGD cases are shown in Figs. 1, 2, 3, and 4, Supplementary Fig. 3, and Supplementary Table 2. All of these cases had Gallyas-positive AT8-positive AGs not only in the limbic system but also in the basal ganglia, brain stem nuclei, and middle frontal, motor, parietal, and occipital cortices (Figs. 1G, 2A, C, E, G, 3G, H, J, 4E, F, H, K, Supplementary Fig. 3A, B, H). AT8-positive GFAs were also noted in the limbic region, striatum, frontal cortex, primary motor cortex, insular cortex, parahippocampal gyrus, and to a lesser degree, in the subthalamic nucleus and inferior olivary nucleus (Figs. 2H, 3H, I, 4G, I, N). AT8-positive NFTs were often scattered in the basal ganglia and brain stem nuclei (Figs. 2A, E, 4K, L, M, O, Supplementary Fig. 3I, K, L).

**Fig. 5:**
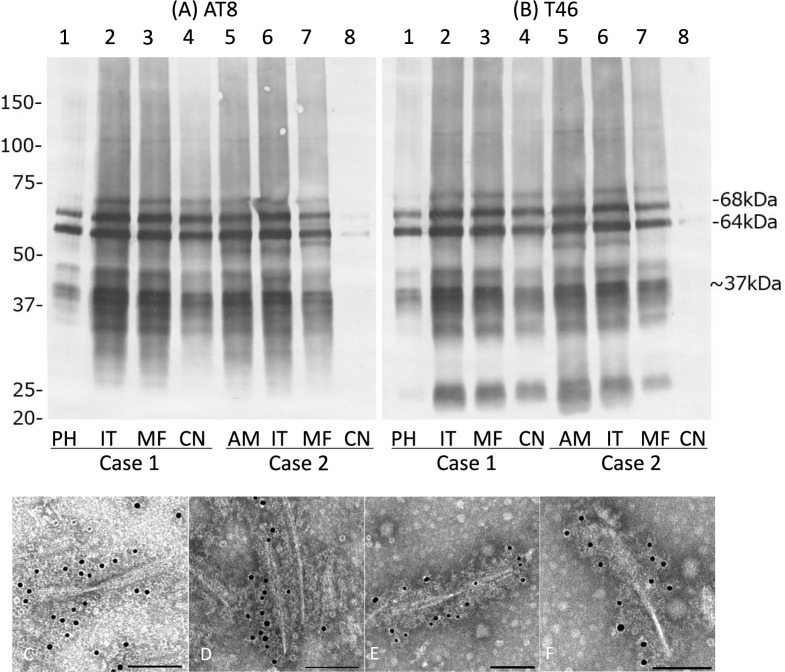
Tau immunoblot and immunoelectron microscopy of the sarkosyl-insoluble, urea-soluble fraction from brains in diffuse form pAGD cases. **A**, **B** Tau immunoblot analysis in cases 1 and 2 with AT8 (**A**) and T46 (**B**). Approximately 68- and 64-kDa bands were noted in all regions examined in cases 1 and 2. Low molecular weight tau fragments of approximately 22-kDa band and 37-kDa doublet were also noted in both cases. Weak 60-kDa bands that suggest tau accumulation as AD pathology were noted in the amygdala and inferior temporal gyrus in case 2. **C**–**F** Immunoelectron microscopy of sarkosyl-insoluble tau from brains in cases 1 (**C**, **D**) and 2 (**E**, **F**). AT8 was used as a primary antibody and was labeled by secondary antibody conjugated to 15-nm gold particles. AT8-positive twisted ribbon-like filaments were noted in both cases. PH: parahippocampal gyrus; IT: inferior temporal gyrus; MF: middle frontal gyrus; CN: caudate nucleus; AM: amygdala

Immunoblot analysis of the sarkosyl-insoluble, urea-soluble fraction prepared from the right hemisphere with AT8 (Fig. 5A) and T46 (Fig. 5B) of cases 1 and 2 for which frozen tissue was available showed approximately 68- and 64-kDa bands and low molecular weight tau fragments of an approximately 22-kDa band and a 37-kDa doublet, being consistent with a pattern that was considered to be a band pattern for AGD [60]. Immunoelectron microscopy of sarkosyl-insoluble fractions from cases 1 and 2 demonstrated twisted ribbon-like filaments labeled with AT8, which are common in AGD brains (Figs. 5C–F) [60].

### Quantity of AGs in each region of all pAGD cases

The quantities of AGs in each anatomical region in diffuse form pAGD cases as well as the other pAGD cases are shown in Table 2. In all pAGD cases, AGs were most frequently noted in the ambient gyrus and CA1 (100.0%, respectively), followed by the amygdala (96.2%), transentorhinal cortex (85.2%), lateral occipitotemporal gyrus (81.8%), subiculum (66.7%), the superior temporal gyrus at the level of the temporal pole (60.0%), insular cortex (43.3%), and inferior temporal gyrus (33.3%). The frequency of cases having AGs in the middle frontal gyrus, primary motor cortex, inferior parietal lobule, peristriate region, caudate nucleus, putamen, periaqueductal gray, substantia nigra, pontine tegmentum, and medulla oblongata ranged from 10 to 20%.

### Severity of neuronal loss in each region in all pAGD and control cases

**Fig. 6:**
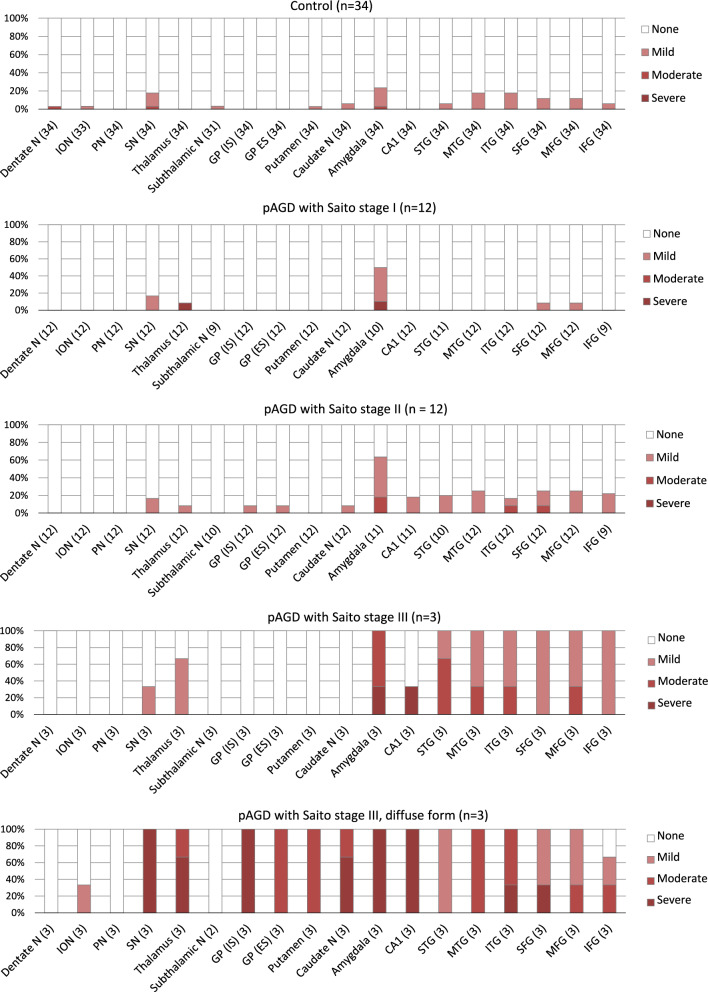
Distribution of neuronal loss with gliosis in pAGD and control cases. In pAGD cases with Saito AG stages I–III, neuronal loss sequentially became more evident with the progression of AGD especially in the amygdala, hippocampal CA1, neocortex, thalamus, and substantia nigra. In diffuse form pAGD cases, evident neuronal loss with gliosis was additionally noted in the caudate nucleus, putamen, and globus pallidus but not in the subthalamic nucleus

In pAGD cases, the frequency and severity of neuronal loss with gliosis was increased with the progression of Saito AG stage in the amygdala, hippocampal CA1, temporo-frontal cortex, substantia nigra, and thalamus (Fig. 6). In control cases, neuronal loss was almost minimal in the neocortex and limbic region. The distribution and severity of neuronal loss in control cases were comparable with those in pAGD cases with Saito AG stage I, although neuronal loss in the amygdala tended to be more evident in the latter group.

In diffuse form pAGD cases, severe neuronal loss with gliosis was consistently noted in the amygdala, ambient gyrus, and ventral portion of the insular cortex (Supplementary Table 2). Neuronal loss with gliosis in the temporal cortex was severe to moderate, while neurons in the frontal cortex were relatively spared. Further, the caudate nucleus, putamen, and thalamus showed severe to moderate neuronal loss (Fig. 2B). The globus pallidus was also consistently affected by severe neuronal loss, which was more prominent in the internal segment (Figs. 1H–L, 4A–E, Supplementary Fig. 3C–G). Tissue degeneration was not noted in the subthalamic nucleus in diffuse form pAGD cases (Figs. 2C, 6). In the brain stem nuclei, severe neuronal loss was consistently noted in the substantia nigra (Figs. 2D, 4J, Supplementary Fig. 3J). The other brain stem nuclei were well spared.

Hippocampal sclerosis characterized by severe neuronal loss with gliosis in the hippocampal CA1 and subiculum was noted in four pAGD cases with Saito AG stage III including all three diffuse form pAGD cases (Fig. 2F and Supplementary Fig. 3M). Among these four pAGD cases with hippocampal sclerosis, three cases (including two diffuse form cases) had LATE-NC. Of remaining 26 pAGD cases, five cases had LATE-NC but lacked hippocampal sclerosis.

### Correlation between Braak NFT stage, Thal phase, Saito AG stage, GFA stage, LATE-NC stage, and the severity of neuronal loss in all pAGD cases

Spearman’s rank-order correlation test demonstrated that the Saito AG stage was significantly correlated the severity of neuronal loss in all regions examined including the limbic system, neocortex, and subcortical nuclei (Supplementary Table 3). The LATE-NC stage was also significantly correlated with the severity of neuronal loss in the entorhinal cortex, hippocampal CA1, subiculum, insular cortex, temporal cortex, frontal cortex, caudate nucleus, and globus pallidus. The GFA stage, age at death, Braak stage, or Thal phase was not significantly correlated with the severity of neuronal loss in any region examined.

### Multivariate analysis of predictors for neuronal loss in all pAGD and control cases

In multivariate ordered logistic regression analyses (independent variables: the age at death, Braak NFT stage, Saito AG stage, LATE-NC stage, and a dependent variable: the severity of neuronal loss), Saito AG stage had an independent effect on neuronal loss in the amygdala, entorhinal cortex, hippocampal CA1, lateral occipitotemporal gyrus, inferior temporal gyrus, middle temporal gyrus, superior temporal gyrus, insular cortex, cingulate gyrus, middle frontal gyrus, orbital gyrus, and substantia nigra (Table 3). The age at death had an independent effect on neuronal loss in the amygdala, and the LATE-NC stage had an independent effect on neuronal loss in the lateral occipitotemporal gyrus, inferior temporal gyrus, and middle temporal gyrus, respectively. In contrast, neither Braak NFT stage nor LATE-NC stage was a significant independent factor of neuronal loss in these regions in our series with Braak NFT stage IV or under.

**Table 3:** Multivariate analyses of predictors for neuronal loss in limbic, neocortical, and subcortical regions

	Odds ratio	95% CI	*p* value
*Multivariate ordered logistic regression analyses*			
Amygdala (n = 63)^a^			
Age at death	1.07	1.01–1.14	0.0179*
Braak NFT stage	0.67	0.35–1.29	0.2320
Saito AG stage	3.18	1.73–5.84	< 0.001**
LATE-NC stage	0.62	0.27–1.44	0.2659
Entorhinal cortex (n = 63)^a^			
Age at death	1.01	0.98–1.05	0.3961
Braak NFT stage	0.82	0.42–1.60	0.5656
Saito AG stage	2.38	1.29–4.39	0.0056**
LATE-NC stage	1.94	0.89–4.22	0.0948
CA1 in the hippocampus^a^			
Age at death	0.76	0.55–1.05	0.0920
Braak NFT stage	5.37	0.50–57.69	0.1656
Saito AG stage	220.70	1.50–32565.95	0.0342*
LATE-NC stage^b^	138.28	0.74–25,781.42	0.0646
Lateral occipitotemporal gyrus (n = 63)^a^			
Age at death	1.00	0.96–1.03	0.8281
Braak NFT stage	0.42	0.17–1.04	0.0619
Saito AG stage	2.82	1.39–5.71	0.0040**
LATE-NC stage	3.93	1.43–10.85	0.0082**
Inferior temporal gyrus (n = 63)^c^			
Age at death	1.01	0.92–1.12	0.8326
Braak NFT stage	0.41	0.14–1.17	0.0951
Saito AG stage	2.88	1.36–6.11	0.0059**
LATE-NC stage	2.97	1.01–8.77	0.0485*
Middle temporal gyrus (n = 63)^a^			
Age at death	1.00	0.91–1.11	0.9727
Braak NFT stage	0.52	0.20–1.37	0.1854
Saito AG stage	2.92	1.34–6.33	0.0068**
LATE-NC stage	3.74	1.24–11.24	0.0190*
Superior temporal gyrus (n = 60)^a^			
Age at death	1.03	0.92–1.16	0.6320
Braak NFT stage	0.56	0.20–1.56	0.2656
Saito AG stage	6.74	2.16–21.02	0.0010**
LATE-NC stage	0.83	0.25–2.73	0.7634
Insular cortex (n = 62)^a^			
Age at death	0.99	0.95–1.04	0.8149
Braak NFT stage	0.73	0.32–1.68	0.4615
Saito AG stage	4.28	1.86–9.84	< 0.001**
LATE-NC stage	2.15	0.82–5.59	0.1179
Cingulate gyrus (n = 60)^a^			
Age at death	1.01	0.92–1.12	0.7919
Braak NFT stage	0.70	0.30–1.63	0.4052
Saito AG stage	2.75	1.27–5.95	0.0100**
LATE-NC stage	1.94	0.72–5.21	0.1891
Middle frontal gyrus (n = 63)^a^			
Age at death	0.96	0.87–1.07	0.4509
Braak NFT stage	0.52	0.20–1.38	0.1903
Saito AG stage	5.44	2.11–14.01	< 0.001**
LATE-NC stage	1.37	0.47–3.96	0.5626
Orbital gyrus (n = 53)^a^			
Age at death	0.97	0.87–1.07	0.5166
Braak NFT stage	0.45	0.17–1.23	0.1208
Saito AG stage	5.84	2.21–15.43	< 0.001**
LATE-NC stage	1.80	0.55–5.95	0.3326
Substantia nigra (n = 63)^a^			
Age at death	0.94	0.84–1.04	0.1985
Braak NFT stage	0.73	0.28–1.88	0.5114
Saito AG stage	2.76	1.25–6.09	0.0116*
LATE-NC stage	1.94	0.67–5.60	0.2230
*Binomial logistic regression analysis*			
Caudate nucleus (n = 63)^d^			
Age at death	0.87	0.75–1.02	0.0821
Moderate NFTs^e^	1.71	0.07–39.80	0.7393
Severe AGs^f^	20.02	1.29–310.27	0.0321*
Moderate LATE-NC^g^	53.87	1.28–2262.10	0.0366*
Putamen (n = 63)^d^			
Age at death	0.84	0.67–1.05	0.1206
Moderate NFTs^e^	0.10	0.00–17.44	0.3783
Severe AGs^f^	473.03	2.55–87,745.07	0.0208*
Moderate LATE-NC^g^	68.84	0.22–21,175.06	0.1477
Globus pallidus (n = 63)^d^			
Age at death	0.98	0.83–1.16	0.8167
Moderate NFTs^e^	0.11	0.00–6.49	0.2895
Severe AGs^f^	85.90	3.24–2277.13	0.0077**
Moderate LATE-NC^g^	6.82	0.15–317.26	0.3270

30 pAGD cases and 34 control cases were examined.

*CI* confidence interval, *NFT* neurofibrillary tangle, *AG* argyrophilic grains, *LATE-NC* limbic-predominant age-related TDP-43 encephalopathy neuropathologic change.

^a^The dependent variable was a four-point staging system of neuronal loss (none, mild, moderate, and severe. The definitions are noted in the text) in each region. The age at death, Braak NFT stage, Saito AG stage, and LATE-NC stage were submitted as independent variables.

^b^LATE-NC stages 1–3 were combined.

^c^Neuronal loss stages 2 and 3 were combined.

^d^The dependent variable was the presence or absence of neuronal loss (stages 0 or 1–3) in each region.

^e^Braak NFT stages III–IV.

^f^Because all diffuse form pAGD cases fit the criteria of Saito AG stage III, diffuse form was regarded as Saito AG stage III in statistical analyses.

^g^LATE-NC stages 2. No pAGD or control case had LATE-NC in stage 3.

*: *p* < 0.05, **: *p* < 0.01

In binomial logistic regression analyses (independent variables: the age at death, moderate NFTs [Braak NFT stages III-IV], severe AGs [Saito AG stages III including diffuse form], and moderate LATE-NC stage [LATE stage 2. No case had LATE-NC with stage 3]. A dependent variable: the presence of neuronal loss in the caudate nucleus, putamen, and globus pallidus, respectively), severe AGs had an independent effect on neuronal loss in the caudate nucleus, putamen, and globus pallidus, respectively (Table 3). Moderate LATE-NC also had an independent effect on neuronal loss in the caudate nucleus (Table 3).

After these analyses, we also submitted the status of GFAs (the GFA stage or presence or absence of GFA) as an independent variable into the model of the amygdala, middle frontal gyrus, caudate nucleus, and putamen. However, the results were not changed (Supplementary Table 4).

### Univariate and multivariate analyses of predictors for dementia in all pAGD and control cases

To examine the effect of pAGD on the development of dementia, we selected 51 cases (23 pAGD cases and 28 control cases) for which data regarding dementia were available and which lacked one or more large infarctions and/or two or more lacunae. Of the 51 cases, 15 cases showed dementia (Table 4). Of the 15 cases with dementia, 13 cases had pAGD (86.7%), and two control cases (13.3%) had only NFTs with Braak stages II or III. The latter two control cases with dementia were clinically diagnosed as chronic schizophrenia, which can cause severe cognitive impairment in the later stage of the clinical course regardless of degenerative changes [13, 46]. Of the remaining 36 cases without dementia, 10 had pAGD (27.8%) and 26 cases had only mild to moderate NFTs in Braak stages I–IV with or without Aβ deposits in Thal phases 1–2 (72.2%). Regarding the effect of chronic schizophrenia, which can cause cognitive impairment, the frequency of schizophrenia in cases with dementia (3 of 15 cases, 20.0%) was significantly lower than that in cases without dementia (25 of 36 cases, 69.4%) (*p* = 0.0018, Fisher’s exact test).

**Table 4:**
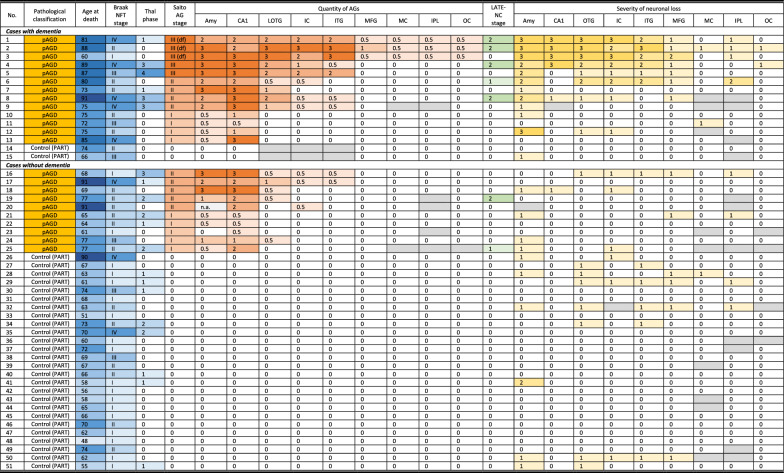
Pathological features in 23 pAGD and 28 control cases with and without dementia

To minimize the influence of vascular lesions on the development of dementia, cases that had one or more large infarctions and/or two or more lacunae in any region in the neocortex or subcortical nuclei were excluded.

*pAGD* pure argyrophilic grain disease, *PART* primary age-related tauopathy, *NFT* neurofibrillary tangle, *AGs* argyrophilic grains, *LATE-NC* limbic-predominant age-related TDP-43 encephalopathy neuropathological change (LATE-NC), *Amy* amygdala, *LOTG* lateral occipitotemporal gyrus, *IC* insular cortex, *ITG* inferior temporal gyrus, *MFG* middle frontal gyrus, *MC* motor cortex, *IPL* inferior parietal lobule, *OC* occipital cortex

Results in univariate analyses using binomial logistic regression models are shown in Supplementary Table 5. The age at death, Braak stages III-IV, Saito AG stage II or higher, presence of AGs in the amygdala, CA1, lateral occipitotemporal gyrus, inferior temporal gyrus, and insular cortex, respectively, moderate and high densities of AGs (50–99 AGs and 100 or more AGs per × 400 visual field) in the amygdala, the high density of AGs in the CA1 (100 or more AGs per × 400 visual field), the presence of LATE-NC, and the presence of moderate to severe neuronal loss in the amygdala had a potential effect on the development of dementia.

Results in multivariate binomial logistic regression analyses with the age at death, Braak NFT stages III–IV, pathological variables regarding AGs in the amygdala, hippocampal CA1, and neocortex, and the presence of LATE-NC as independent variables are shown in Table 5. One hundred or more AGs per × 400 visual field in the amygdala, 100 or more AGs per × 400 visual field in the hippocampus CA1, and the presence of one or more AGs in the inferior temporal gyrus had an independent effect on the development of dementia. Further, the presence of either 100 or more AGs per × 400 visual field in the amygdala or CA1 or the presence of AGs in the inferior temporal gyrus also had an independent effect on the development of dementia.

**Table 5:** Multivariate binomial logistic regression analyses of predictors for dementia in pAGD and control cases

	OR	95% CI	*p*
*Quantity of AGs in the amygdala*			
Model 1 (n = 50)			
Age at death	1.09	0.98–1.22	0.1122
Braak NFT stages III-IV	2.08	0.34–12.79	0.4280
One to 49 AGs in the amygdala (per × 400 visual field)	1.57	0.30–8.31	0.5928
Presence of LATE-NC	2.51	0.27–23.78	0.4221
Model 2 (n = 50)			
Age at death	1.09	0.98–1.22	0.1159
Braak NFT stages III-IV	1.74	0.26–11.74	0.5690
50–99 AGs in the amygdala (per × 400 visual field)	2.43	0.17–34.96	0.5139
Presence of LATE-NC	2.03	0.18–23.19	0.5689
Model 3 (n = 50)			
Age at death	1.07	0.95–1.20	0.2394
Braak NFT stages III-IV	3.55	0.46–27.14	0.2224
100 or more AGs in the amygdala (per × 400 visual field)	10.02	1.12–89.43	0.0391*
Presence of LATE-NC	3.67	0.32–42.61	0.2985
*Quantity of AGs in the hippocampal CA1*			
Model 4 (n = 51)			
Age at death	1.07	0.97–1.17	0.1669
Braak NFT stages III-IV	3.08	0.55–17.23	0.1998
One to 49 AGs in the CA1 (per × 400 visual field)	3.63	0.56–23.50	0.1754
Presence of LATE-NC	5.13	0.57–45.82	0.1432
Model 5 (n = 51)			
Age at death	1.07	0.97–1.18	0.1913
Braak NFT stages III-IV	2.83	0.49–16.29	0.2441
20–99 AGs in the CA1 (per × 400 visual field)	0.76	0.08–7.03	0.8054
Presence of LATE-NC	4.55	0.41–50.60	0.2176
Model 6 (n = 51)			
Age at death	1.07	0.97–1.17	0.1704
Braak NFT stages III-IV	2.19	0.36–13.34	0.3954
100 or more AGs in the CA1 (per × 400 visual field)	12.22	1.70–87.81	0.0128*
Presence of LATE-NC	5.43	0.35–83.90	0.2258
*Presence of AGs in the neocortex*			
Model 7 (n = 49)			
Age at death	1.06	0.95–1.18	0.2686
Braak NFT stages III-IV	2.03	0.27–15.20	0.4890
Presence of AGs in the lateral occipitotemporal gyrus	5.74	0.97–34.07	0.0544
Presence of LATE-NC	2.05	0.18–24.03	0.5673
Model 8 (n = 49)			
Age at death	1.06	0.96–1.18	0.2484
Braak NFT stages III-IV	1.61	0.19–13.75	0.6622
Presence of AGs in the inferior temporal gyrus	8.18	1.03–65.13	0.0471*
Presence of LATE-NC	3.33	0.28–38.96	0.3377
*Combination of AGs in the amygdala, CA1, or neocortex*			
Model 9 (n = 49)			
Age at death	1.04	0.92–1.17	0.5015
Braak NFT stages III-IV	1.82	0.17–19.62	0.6225
Either 100 or more AGs per × 400 visual field in the amygdala or CA1 or the presence of AGs in the inferior temporal gyrus	13.92	2.12–91.49	0.0061**
Presence of LATE-NC	5.72	0.39–83.79	0.2025

51 cases (23 pAGD cases and 28 control cases) that lacked two or more lacunae or larger infarctions in the cortex and/or subcortical regions were examined using multivariate analyses.

*pAGD* pure argyrophilic grain disease, *OR* odds ratio, *CI* confidence interval, *NFT* neurofibrillary tangle, *AG* argyrophilic grains, *LATE-NC* limbic-predominant age-related TDP-43 encephalopathy neuropathologic change.

*: *p* < 0.05, **: *p* < 0.01

## Discussion

This is the first study demonstrating that AGs have an independent effect on neuronal loss in the amygdala, hippocampal CA1, temporo-frontal cortex, striatum, globus pallidus, and substantia nigra, as well as on the development of dementia, in cases with a low to moderate Braak NFT stage (I to IV). The main findings are: (1) In 30 pAGD cases with Saito AG stages I-III, neuronal loss first developed in the amygdala, followed by the temporo-frontal cortex and substantia nigra, and finally the striatum and globus pallidus, with the progression of the Saito AG stage. Of 30 pAGD cases, three were classified as diffuse form pAGD, having evident neuronal loss in the striatum, globus pallidus, substantia nigra, hippocampal CA1, and frontal cortex, in addition to the amygdala and adjacent temporal cortex. (2) In multivariate analyses with 30 pAGD and 34 control cases, the Saito AG stage had an effect independent of the age at death, Braak NFT stage, or LATE-NC stage on neuronal loss in the amygdala, entorhinal cortex, hippocampal CA1, temporal cortex, middle frontal gyrus, orbital gyrus, cingulate gyrus, insular cortex, and substantia nigra. The presence of severe AGs with Saito AG stage III also had an independent effect on neuronal loss in the caudate nucleus, putamen, and globus pallidus. (3) In multivariate analyses of 23 pAGD and 28 control cases that lacked significant vascular lesions, 100 or more AGs per × 400 visual field in the amygdala, 100 or more AGs per × 400 visual field in the hippocampal CA1, and the presence of AGs in the inferior temporal gyrus had an effect independent of the age, NFTs, or LATE-NC on the development of dementia.

It has been reported that some cases with AGs show severe atrophy or degeneration in the amygdala [22, 51, 52, 55, 56, 68]. However, the present study first revealed an independent effect of the Saito AG stage on neuronal loss in the hippocampal CA1 as well. This result is consistent with several previous findings regarding hippocampal atrophy in AGD cases with Saito AG stage III [1]. Although findings regarding the relationship between hippocampal degeneration and LATE-NC have been accumulated [23, 28, 40, 42], based on our findings, the effect of AGs, besides LATE-NC, on neuronal loss in the hippocampal CA1 should be also considered when examining the hippocampal atrophy and memory impairment in cases with a low to moderate Braak NFT stage.

In a previous study reported by Saito et al. [53], it was noticed that the distribution of AGs corresponding to their stage III was closely associated with the development of dementia. Interestingly, their view was supported by our results in multivariate analyses. For example, in our study, the presence of 100 or more AGs per × 400 visual field in the amygdala and the presence of 100 or more AGs per × 400 visual field in the hippocampal CA1 were independent factors in the development of dementia, while lower density of AGs in these regions was not (Table 5). Likewise, the presence of AGs in the inferior temporal gyrus was an independent factor on the occurrence of dementia, while the presence of AGs in the lateral occipitotemporal gyrus was not (Table 5). These statuses, which had independent effects on dementia, are consistent with the pathological statuses of Saito AG stage III. Further, this potential relationship between AGs and dementia may be supported by our finding that the Saito AG stage had an independent effect on neuronal loss not only in the limbic system but also in widespread regions of the neocortex (Table 3, Fig. 6). Given these findings, evaluating whether there are 100 or more AGs per × 400 visual field either in the amygdala or hippocampal CA1 and evaluating whether there are AGs in the inferior temporal gyrus might be useful to infer whether AGs contributed at least partially to the occurrence of dementia in each patient.

In contrast to our study, several previous studies failed to demonstrate the potential relationship between AGs and cognitive impairment [17, 41, 48, 50]. However, the procedures of the pathological assessment of AGs in previous studies were varied. In several studies, only limbic regions (e.g., the amygdala, entorhinal cortex, subiculum, and/or hippocampal CA1) but not the neocortex were examined [41, 48, 50]. Regarding the data on AGs, the presence/absence data [17, 48], semiquantitative data [41, 50], or Saito AG stage [51] were used in statistical analyses. Further, the pathological backgrounds in the series examined were also varied: in most previous studies in which a relationship between AGs and cognitive impairment was not revealed, AGD cases having severe NFTs (Braak NFT stages V–VI) were not excluded from the analyses [41, 48, 50]. It is noteworthy that, in all studies (including ours) in which cases having AD, other degenerative changes, and significant vascular lesions were excluded, the relationship between AGs and dementia was observed [6, 51]. In a previous study, based on the fact that none of eight AGD cases with dementia had NFTs with low Braak stages of 0 to II, it was concluded that AGs may be an additive pathology that may reduce the threshold for dementia [22]. However, in our series, 6 of 13 pAGD cases with dementia (46.2%) had NFTs with Braak stages I–II (Table 4) and multivariate analyses demonstrated an effect of AGs independent of Braak stage on dementia (Table 5). Given these findings, when the proportion of cases bearing the high density of AGs in the limbic system, the proportion of cases bearing AGs in the inferior temporal gyrus, and/or the proportion of cases with a low to moderate Braak NFT stage in a series examined are small, the effect of AGs on cognitive function may be difficult to detect. In addition, the staining method to reveal AGs may also have some effect on the analysis of the relationship between AGs and cognitive function. For example, because AT8 immunohistochemistry can reveal AGs more sensitively than the Gallyas method [45], it is possible that the threshold for the occurrence of dementia due to AGs may appear to be increased in a study using AT8 immunohistochemistry than in a study using the Gallyas method. This view can be inferred from a report that, although AT8 can reveal very early changes of NFTs, no case having only such lesions showed dementia clinically [10].

In our series, mild to moderate numbers of AGs (i.e., less than 100 AGs per × 400 visual field in the amygdala, less than 100 AGs per × 400 visual field in the hippocampal CA1) or not so widely distributed AGs (i.e., the presence of AGs in the lateral occcipitotemporal gyrus) were not associated with dementia. However, it does not necessarily suggest that mild to moderate AGs do not affect cognitive function and psychiatric status at all. For example, in a recent study in which 1449 serial forensic autopsy cases were examined, the frequencies of suicide in AGD cases with each Saito AG stage (stages I, II, or III) were significantly higher than in cases without AGs, respectively [69]. Likewise, although the frequency of AGs was reported to be significantly higher in cases who showed late-onset psychotic disorder without dementia than that in age-matched control cases, AGs in all cases of late-onset psychotic disorder remained in Saito AG stages I or II [39]. These findings support the possibility that mild to moderate AGs may be associated with some psychiatric conditions lacking dementia. Further data regarding the impact of mild to moderate AGs on cognitive function and psychiatric status should be accumulated.

Multivariate analyses in this study demonstrated that Saito AG stage had an independent effect on neuronal loss in the striatum, globus pallidus, and substantia nigra (Table 3). Indeed, in our three diffuse-form pAGD cases, moderate to severe neuronal loss was consistently noted in the striatum, globus pallidus, and substantia nigra (Fig. 6), and two of three cases clinically showed asymmetric parkinsonism in the later clinical course. Likewise, among previously reported AGD cases that had AGs in the basal ganglia and/or brain stem, some cases showed parkinsonism (Supplementary Table 6) [4, 16, 19–21, 35, 62]. The detailed pathophysiological background and frequency of motor disturbances in pAGD cases need to be explored in the future.

Limitations of this study are as follows: (1) the numbers of pAGD cases and control cases examined were small, so that some sampling bias might affect our results. However, the significant relationship between AGs and cognitive impairment observed in our study was consistent with those in two independent autopsy series [6, 53] in which AD cases, other degenerative disease cases, and cases with vascular changes were excluded. (2) In our study, probably because we extracted pAGD cases that lacked the other degenerative diseases, the frequency of cases with LATE-NC in our series was relatively low. This may be because the effect of LATE-NC on neuronal loss in the hippocampal CA1 failed to be demonstrated. However, this potential sampling bias does not deny the association of AGs with neuronal loss as well as dementia. (3) In the present study, the presence of 100 or more AGs per × 400 visual field in the amygdala or hippocampal CA1 and the presence of AGs in the inferior temporal gyrus were factors independent of the occurrence of dementia. However, the number of AGs as the threshold for dementia may be affected by the staining method employed because AT8 immunohistochemistry is more sensitive for the detection of AGs than the Gallyas method [45]. (4) In this study, the presence of dementia was retrospectively determined based on the clinical summary, chart review, and an interview of physicians who provided long-term treatment in the late stage of the clinical course. However, this information was obtained before the pathological examination, so that the clinical assessment by each physician at least was not influenced by pathological findings. In addition, because the diagnosis of dementia was made according to the DSM-III-R regardless of cause in this study, the dementia state might be associated with psychiatric disorders rather than neurodegenerative diseases in some cases. However, because the frequency of schizophrenia in our cases with dementia was significantly lower than that in our cases without dementia (20.0% vs. 69.4%), it is plausible that dementia in our series cannot be explained by a past history of schizophrenia.

In conclusion, in our series with low to moderate Braak stages, the progression of AGs had an independent effect on neuronal loss in the limbic region, temporo-frontal cortex, and subcortical nuclei. Further, 100 or more Gallyas-positive AGs per × 400 visual field in the amygdala or hippocampal CA1 and the progression of Gallyas-positive AGs to the inferior temporal cortex were factors independent of dementia. These findings suggest that the effect of AGs should be considered when examining the pathogenic backgrounds of limbic and neocortical atrophy and cognitive impairment, at least in cases with a low to moderate Braak stage.

### Supplementary Information

Supplementary figure 1. Representative photos of the grading system (stages 0–4) of neuronal loss with gliosis in the cerebral cortex. **A**, **E** Stage 0. No neuronal loss. **B**, **F** Stage 1. Mild neuronal loss. **C**, **G** Stage 2. Moderate neuronal loss. **D**, **H** Stage 3. Severe neuronal loss. **A**–**D** H&E stain, **E**–**H** KB stain. Scale bars: **A**–**D** 200 μm, **E**–**H** 200 μm. The most severe grade was recorded as the stage in each region. Details of the definition of each stage are noted in the text.

Supplementary figure 2. Representative photos of the grading system (stages 0–4) of neuronal loss with gliosis in the subcortical nuclei. **A**, **E**, **I**, **M** Stage 0. No neuronal loss. **B**, **F**, **J**, **N** Stage 1. Mild neuronal loss. **C**, **G**, **K**, **O** Stage 2. Moderate neuronal loss. **D**, **H**, **L**, **P** Stage 3. Severe neuronal loss. **A**–**H** H&E stain, **I**–**P** KB stain. Scale bars: **A**–**D**, **I**–**L** 100 μm, **E**–**H**, **M**–**P** 50 μm. The most severe grade was recorded as the stage in each region. Details of the definition of each stage are noted in the text.

Supplementary figure 3. Pathological findings in case 3. **A** 4R tau-positive AGs in the amygdala. Scale bar = 30 μm. Inset: Argyrophilic grains in the same region. Gallyas method. Scale bar = 10 μm. **B** 4R tau-positive Betz cell and argyrophillic grains in the primary motor cortex. Scale bar = 30 μm. Inset: The Gallyas method did not demonstrate argyrophilia in Betz cells. Scale bar = 10 μm. **C** Severe neuronal loss with gliosis in the globus pallidus. Tissue degeneration is more evident in the dorsal portion of the site. H&E stain. Scale bar = 200 μm. **D** Holzer stain demonstrated severe gliosis in the dorsal portion rather than the ventral portion in the globus pallidus. Scale bar = 200 μm. **E**, **F** Severe neuronal loss and gliosis in the globus pallidus demonstrated by H&E stain (**E**) and GFAP immunohistochemistry (**F**). Scale bar = 20 μm. **G** AT8-positive neurons and threads in the globus pallidus. Scale bar = 20 μm. **H** AGs in the caudate nucleus. Gallyas method. Scale bar = 50 μm. **I** AT8-positive NFTs and threads in the putamen. Scale bar = 20 μm. **J** Severe neuronal loss in the substantia nigra. H&E stain. Scale bar = 600 μm. **K** AT8-positive NFTs and threads in the substantia nigra. Scale bar = 20 μm. **L** AT8-positive glial cells and fine threads in the white matter in the cerebellum. Scale bar = 20 μm. **M** Hippocampal sclerosis characterized by severe loss in the CA1 and subiculum. H&E stain. Scale bar = 500 μm.

Supplementary file 1

Supplementary tables

## Data Availability

All data of this study are provided in the Tables and Supplementary Table.

## References

[1] Adachi T, Saito Y, Hatsuta H, Funabe S, Tokumaru AM, Ishii K, Arai T, Sawabe M, Kanemaru K, Miyashita A, Kuwano R, Nakashima K, Murayama S (2010) Neuropathological asymmetry in argyrophilic grain disease. J Neuropathol Exp Neurol 69:737–744. 10.1097/NEN.0b013e3181e5ae5c 20535032 10.1097/NEN.0b013e3181e5ae5c

[2] Ahmed Z, Bigio EH, Budka H, Dickson DW, Ferrer I, Ghetti B, Giaccone G, Hatanpaa KJ, Holton JL, Josephs KA, Powers J, Spina S, Takahashi H, White CL 3rd, Revesz T, Kovacs GG (2013) Globular glial tauopathies (GGT): consensus recommendations. Acta Neuropathol 126:537–544. 10.1007/s00401-013-1171-0 23995422 10.1007/s00401-013-1171-0 PMC3914659

[3] American Psychiatric Association (1987) Diagnostic and statistical manual of mental disorders, 3rd edn revised (DSM-III-R). American Psychiatric Association, Washington DC

[4] Arakawa A, Goto R, Higashihara M, Hiroyoshi Y, Shioya A, Hara M, Orita M, Matsubara T, Sengoku R, Kameyama M, Tokumaru AM, Hasegawa M, Toda T, Iwata A, Murayama S, Saito Y (2024) Clinicopathological study on dementia with grains presenting with parkinsonism compared with a typical case. Neuropathology. 10.1111/neup.12973 38558069 10.1111/neup.12973

[5] Botez G, Probst A, Ipsen S, Tolnay M (1999) Astrocytes expressing hyperphosphorylated tau protein without glial fibrillary tangles in argyrophilic grain disease. Acta Neuropathol 98:251–256. 10.1007/s004010051077 10483782 10.1007/s004010051077

[6] Braak H, Braak E (1987) Argyrophilic grains: characteristic pathology of cerebral cortex in cases of adult onset dementia without Alzheimer changes. Neurosci Lett 76:124–127. 10.1016/0304-3940(87)90204-7 2438598 10.1016/0304-3940(87)90204-7

[7] Braak H, Braak E (1998) Argyrophilic grain disease: frequency of occurrence in different age categories and neuropathological diagnostic criteria. J Neural Transm (Vienna) 105:801–819. 10.1007/s007020050096 9869320 10.1007/s007020050096

[8] Braak H, Del Tredici K, Rüb U, de Vos RA, Jansen Steur EN, Braak E (2003) Staging of brain pathology related to sporadic Parkinson’s disease. Neurobiol Aging 24:197–211. 10.1016/s0197-4580(02)00065-9 12498954 10.1016/s0197-4580(02)00065-9

[9] Braak H, Alafuzoff I, Arzberger T, Kretzschmar H, Del Tredici K (2006) Staging of Alzheimer disease-associated neurofibrillary pathology using paraffin sections and immunocytochemistry. Acta Neuropathol 112:389–404. 10.1007/s00401-006-0127-z 16906426 10.1007/s00401-006-0127-z PMC3906709

[10] Braak H, Del Tredici K (2011) The pathological process underlying Alzheimer’s disease in individuals under thirty. Acta Neuropathol 121:171–181. 10.1007/s00401-010-0789-4 21170538 10.1007/s00401-010-0789-4

[11] Cairns NJ, Bigio EH, Mackenzie IR, Neumann M, Lee VM, Hatanpaa KJ, White CL 3rd, Schneider JA, Grinberg LT, Halliday G, Duyckaerts C, Lowe JS, Holm IE, Tolnay M, Okamoto K, Yokoo H, Murayama S, Woulfe J, Munoz DG, Dickson DW, Ince PG, Trojanowski JQ, Mann DM, Consortium for Frontotemporal Lobar Degeneration (2007) Neuropathologic diagnostic and nosologic criteria for frontotemporal lobar degeneration: consensus of the Consortium for Frontotemporal Lobar Degeneration. Acta Neuropathol 114:5–22. 10.1007/s00401-007-0237-2 17579875 10.1007/s00401-007-0237-2 PMC2827877

[12] Crary JF, Trojanowski JQ, Schneider JA, Abisambra JF, Abner EL, Alafuzoff I, Arnold SE, Attems J, Beach TG, Bigio EH, Cairns NJ, Dickson DW, Gearing M, Grinberg LT, Hof PR, Hyman BT, Jellinger K, Jicha GA, Kovacs GG, Knopman DS, Kofler J, Kukull WA, Mackenzie IR, Masliah E, McKee A, Montine TJ, Murray ME, Neltner JH, Santa-Maria I, Seeley WW, Serrano-Pozo A, Shelanski ML, Stein T, Takao M, Thal DR, Toledo JB, Troncoso JC, Vonsattel JP, White CL 3rd, Wisniewski T, Woltjer RL, Yamada M, Nelson PT (2014) Primary age-related tauopathy (PART): a common pathology associated with human aging. Acta Neuropathol 128:755–766. 10.1007/s00401-014-1349-0 25348064 10.1007/s00401-014-1349-0 PMC4257842

[13] Dwork AJ, Susser ES, Keilp J, Waniek C, Liu D, Kaufman M, Zemishlany Z, Prohovnik I (1998) Senile degeneration and cognitive impairment in chronic schizophrenia. Am J Psychiatry 155:1536–1543. 10.1176/ajp.155.11.1536 9812114 10.1176/ajp.155.11.1536

[14] Hasegawa M, Arai T, Nonaka T, Kametani F, Yoshida M, Hashizume Y, Beach TG, Buratti E, Baralle F, Morita M, Nakano I, Oda T, Tsuchiya K, Akiyama H (2008) Phosphorylated TDP-43 in frontotemporal lobar degeneration and amyotrophic lateral sclerosis. Ann Neurol 64:60–70. 10.1002/ana.21425 18546284 10.1002/ana.21425 PMC2674108

[15] Hauw JJ, Daniel SE, Dickson D, Horoupian DS, Jellinger K, Lantos PL, McKee A, Tabaton M, Litvan I (1994) Preliminary NINDS neuropathologic criteria for Steele–Richardson–Olszewski syndrome (progressive supranuclear palsy). Neurology 44:2015–2019. 10.1212/wnl.44.11.2015 7969952 10.1212/wnl.44.11.2015

[16] Hokelekli FO, Whitwell JL, Machulda MM, Jones DT, Uitti RJ, Pham NTT, Giannini C, Baker M, Lowe VJ, Dickson DW, Josephs KA (2021) Underlying pathology identified after 20 years of disease course in two cases of slowly progressive frontotemporal dementia syndromes. Neurocase 27:212–222. 10.1080/13554794.2021.1918723 33904372 10.1080/13554794.2021.1918723 PMC8189252

[17] Iida MA, Farrell K, Walker JM, Richardson TE, Marx GA, Bryce CH, Purohit D, Ayalon G, Beach TG, Bigio EH, Cortes EP, Gearing M, Haroutunian V, McMillan CT, Lee EB, Dickson DW, McKee AC, Stein TD, Trojanowski JQ, Woltjer RL, Kovacs GG, Kofler JK, Kaye J, White CL 3rd, Crary JF (2021) Predictors of cognitive impairment in primary age-related tauopathy: an autopsy study. Acta Neuropathol Commun 9:134. 10.1186/s40478-021-01233-3 34353357 10.1186/s40478-021-01233-3 PMC8340493

[18] Ikeda C, Yokota O, Nagao S, Ishizu H, Oshima E, Hasegawa M, Okahisa Y, Terada S, Yamada N (2016) The relationship between development of neuronal and astrocytic tau pathologies in subcortical nuclei and progression of argyrophilic grain disease. Brain Pathol 26:488–505. 10.1111/bpa.12319 26439704 10.1111/bpa.12319 PMC8029468

[19] Inoue K, Sugase S, Naka T, Ikeuchi T, Murayama S, Fujimura H (2023) An autopsy case of diffuse atypical argyrophilic grain disease (AGD) with presenile onset and three-year course of motor and cognitive impairment. Neuropathology. 10.1111/neup.12949 37936523 10.1111/neup.12949

[20] Ishihara K, Araki S, Ihori N, Shiota J, Kawamura M, Yoshida M, Hashizume Y, Nakano I (2005) Argyrophilic grain disease presenting with frontotemporal dementia: a neuropsychological and pathological study of an autopsied case with presenile onset. Neuropathology 25:165–170. 10.1111/j.1440-1789.2005.00598.x 15875911 10.1111/j.1440-1789.2005.00598.x

[21] Itagaki S, McGeer PL, Akiyama H, Beattie BL, Walker DG, Moore GR, McGeer EG (1989) A case of adult-onset dementia with argyrophilic grains. Ann Neurol 26:685–689. 10.1002/ana.410260517 2817845 10.1002/ana.410260517

[22] Josephs KA, Whitwell JL, Parisi JE, Knopman DS, Boeve BF, Geda YE, Jack CR Jr, Petersen RC, Dickson DW (2008) Argyrophilic grains: A distinct disease or an additive pathology? Neurobiol Aging 29:566–573. 10.1016/j.neurobiolaging.2006.10.032 17188783 10.1016/j.neurobiolaging.2006.10.032 PMC2727715

[23] Josephs KA, Dickson DW, Tosakulwong N, Weigand SD, Murray ME, Petrucelli L, Liesinger AM, Senjem ML, Spychalla AJ, Knopman DS, Parisi JE, Petersen RC, Jack CR Jr, Whitwell JL (2017) Rates of hippocampal atrophy and presence of post-mortem TDP-43 in patients with Alzheimer’s disease: a longitudinal retrospective study. Lancet Neurol 16:917–924. 10.1016/S1474-4422(17)30284-3 28919059 10.1016/S1474-4422(17)30284-3 PMC5646369

[24] Kovacs GG, Molnár K, László L, Ströbel T, Botond G, Hönigschnabl S, Reiner-Concin A, Palkovits M, Fischer P, Budka H (2011) A peculiar constellation of tau pathology defines a subset of dementia in the elderly. Acta Neuropathol 122:205–222. 10.1007/s00401-011-0819-x 21437732 10.1007/s00401-011-0819-x

[25] Kovacs GG, Milenkovic I, Wöhrer A, Höftberger R, Gelpi E, Haberler C, Hönigschnabl S, Reiner-Concin A, Heinzl H, Jungwirth S, Krampla W, Fischer P, Budka H (2013) Non-Alzheimer neurodegenerative pathologies and their combinations are more frequent than commonly believed in the elderly brain: a community-based autopsy series. Acta Neuropathol 126:365–384. 10.1007/s00401-013-1157-y 23900711 10.1007/s00401-013-1157-y

[26] Kovacs GG, Xie SX, Robinson JL, Lee EB, Smith DH, Schuck T, Lee VM, Trojanowski JQ (2018) Sequential stages and distribution patterns of aging-related tau astrogliopathy (ARTAG) in the human brain. Acta Neuropathol Commun 6:50. 10.1186/s40478-018-0552-y 29891013 10.1186/s40478-018-0552-y PMC5996526

[27] Lee EB, Porta S, Michael Baer G, Xu Y, Suh E, Kwong LK, Elman L, Grossman M, Lee VM, Irwin DJ, Van Deerlin VM, Trojanowski JQ (2017) Expansion of the classification of FTLD-TDP: distinct pathology associated with rapidly progressive frontotemporal degeneration. Acta Neuropathol 134:65–78. 10.1007/s00401-017-1679-9 28130640 10.1007/s00401-017-1679-9 PMC5521959

[28] Leverenz JB, Agustin CM, Tsuang D, Peskind ER, Edland SD, Nochlin D, DiGiacomo L, Bowen JD, McCormick WC, Teri L, Raskind MA, Kukull WA, Larson EB (2002) Clinical and neuropathological characteristics of hippocampal sclerosis: a community-based study. Arch Neurol 59:1099–1106. 10.1001/archneur.59.7.1099 12117357 10.1001/archneur.59.7.1099

[29] Mackenzie IR, Neumann M, Bigio EH, Cairns NJ, Alafuzoff I, Kril J, Kovacs GG, Ghetti B, Halliday G, Holm IE, Ince PG, Kamphorst W, Revesz T, Rozemuller AJ, Kumar-Singh S, Akiyama H, Baborie A, Spina S, Dickson DW, Trojanowski JQ, Mann DM (2010) Nomenclature and nosology for neuropathologic subtypes of frontotemporal lobar degeneration: an update. Acta Neuropathol 119:1–4. 10.1007/s00401-009-0612-2 19924424 10.1007/s00401-009-0612-2 PMC2799633

[30] Mackenzie IR, Neumann M, Baborie A, Sampathu DM, Du Plessis D, Jaros E, Perry RH, Trojanowski JQ, Mann DM, Lee VM (2011) A harmonized classification system for FTLD-TDP pathology. Acta Neuropathol 122:111–113. 10.1007/s00401-011-0845-8 21644037 10.1007/s00401-011-0845-8 PMC3285143

[31] Mackenzie IR, Frick P, Neumann M (2014) The neuropathology associated with repeat expansions in the* C9ORF72* gene. Acta Neuropathol 127:347–357. 10.1007/s00401-013-1232-4 24356984 10.1007/s00401-013-1232-4

[32] Masliah E, Hansen LA, Quijada S, DeTeresa R, Alford M, Kauss J, Terry R (1991) Late onset dementia with argyrophilic grains and subcortical tangles or atypical progressive supranuclear palsy? Ann Neurol 29:389–396. 10.1002/ana.410290409 1929210 10.1002/ana.410290409

[33] Matsuda H, Mizumura S, Nagao T, Ota T, Iizuka T, Nemoto K, Takemura N, Arai H, Homma A (2007) Automated discrimination between very early Alzheimer disease and controls using an easy Z-score imaging system for multicenter brain perfusion single-photon emission tomography. AJNR Am J Neuroradiol 28:731–736 17416830 PMC7977345

[34] Mattila P, Togo T, Dickson DW (2002) The subthalamic nucleus has neurofibrillary tangles in argyrophilic grain disease and advanced Alzheimer’s disease. Neurosci Lett 320:81–85. 10.1016/s0304-3940(02)00006-x 11849769 10.1016/s0304-3940(02)00006-x

[35] Maurage CA, Sergeant N, Schraen-Maschke S, Lebert F, Ruchoux MM, Sablonnière B, Pasquier F, Delacourte A (2003) Diffuse form of argyrophilic grain disease: a new variant of four-repeat tauopathy different from limbic argyrophilic grain disease. Acta Neuropathol 106:575–583. 10.1007/s00401-003-0762-6 14517683 10.1007/s00401-003-0762-6

[36] McKeith IG, Dickson DW, Lowe J, Emre M, O’Brien JT, Feldman H, Cummings J, Duda JE, Lippa C, Perry EK, Aarsland D, Arai H, Ballard CG, Boeve B, Burn DJ, Costa D, Del Ser T, Dubois B, Galasko D, Gauthier S, Goetz CG, Gomez-Tortosa E, Halliday G, Hansen LA, Hardy J, Iwatsubo T, Kalaria RN, Kaufer D, Kenny RA, Korczyn A, Kosaka K, Lee VM, Lees A, Litvan I, Londos E, Lopez OL, Minoshima S, Mizuno Y, Molina JA, Mukaetova-Ladinska EB, Pasquier F, Perry RH, Schulz JB, Trojanowski JQ, Yamada M, Consortium on DLB (2005) Diagnosis and management of dementia with Lewy bodies: third report of the DLB Consortium. Neurology 65:1863–1872. 10.1212/01.wnl.0000187889.17253.b1 16237129 10.1212/01.wnl.0000187889.17253.b1

[37] Miki T, Yokota O, Haraguchi T, Ishizu H, Hasegawa M, Ishihara T, Ueno SI, Takenoshita S, Terada S, Yamada N (2020) Factors associated with development and distribution of granular/fuzzy astrocytes in neurodegenerative diseases. Brain Pathol 30:811–830. 10.1111/bpa.12843 32293067 10.1111/bpa.12843 PMC7383906

[38] Mirra SS, Heyman A, McKeel D, Sumi SM, Crain BJ, Brownlee LM, Vogel FS, Hughes JP, van Belle G, Berg L (1991) The consortium to establish a registry for Alzheimer’s disease (CERAD). Part II. Standardization of the neuropathologic assessment of Alzheimer’s disease. Neurology 41:479–486. 10.1212/wnl.41.4.479 2011243 10.1212/wnl.41.4.479

[39] Nagao S, Yokota O, Ikeda C, Takeda N, Ishizu H, Kuroda S, Sudo K, Terada S, Murayama S, Uchitomi Y (2014) Argyrophilic grain disease as a neurodegenerative substrate in late-onset schizophrenia and delusional disorders. Eur Arch Psychiatry Clin Neurosci 264:317–331. 10.1007/s00406-013-0472-6 24272318 10.1007/s00406-013-0472-6

[40] Nag S, Yu L, Capuano AW, Wilson RS, Leurgans SE, Bennett DA, Schneider JA (2015) Hippocampal sclerosis and TDP-43 pathology in aging and Alzheimer disease. Ann Neurol 77:942–952. 10.1002/ana.24388 25707479 10.1002/ana.24388 PMC4447563

[41] Nelson PT, Abner EL, Schmitt FA, Kryscio RJ, Jicha GA, Smith CD, Davis DG, Poduska JW, Patel E, Mendiondo MS, Markesbery WR (2010) Modeling the association between 43 different clinical and pathological variables and the severity of cognitive impairment in a large autopsy cohort of elderly persons. Brain Pathol 20:66–79. 10.1111/j.1750-3639.2008.00244.x 19021630 10.1111/j.1750-3639.2008.00244.x PMC2864342

[42] Nelson PT, Schmitt FA, Lin Y, Abner EL, Jicha GA, Patel E, Thomason PC, Neltner JH, Smith CD, Santacruz KS, Sonnen JA, Poon LW, Gearing M, Green RC, Woodard JL, Van Eldik LJ, Kryscio RJ (2011) Hippocampal sclerosis in advanced age: clinical and pathological features. Brain 134:1506–1518. 10.1093/brain/awr053 21596774 10.1093/brain/awr053 PMC3097889

[43] Nelson PT, Dickson DW, Trojanowski JQ, Jack CR, Boyle PA, Arfanakis K, Rademakers R, Alafuzoff I, Attems J, Brayne C, Coyle-Gilchrist ITS, Chui HC, Fardo DW, Flanagan ME, Halliday G, Hokkanen SRK, Hunter S, Jicha GA, Katsumata Y, Kawas CH, Keene CD, Kovacs GG, Kukull WA, Levey AI, Makkinejad N, Montine TJ, Murayama S, Murray ME, Nag S, Rissman RA, Seeley WW, Sperling RA, White CL 3rd, Yu L, Schneider JA (2019) Limbic-predominant age-related TDP-43 encephalopathy (LATE): consensus working group report. Brain 142:1503–1527. 10.1093/brain/awz099 31039256 10.1093/brain/awz099 PMC6536849

[44] Nelson PT, Lee EB, Cykowski MD, Alafuzoff I, Arfanakis K, Attems J, Brayne C, Corrada MM, Dugger BN, Flanagan ME, Ghetti B, Grinberg LT, Grossman M, Grothe MJ, Halliday GM, Hasegawa M, Hokkanen SRK, Hunter S, Jellinger K, Kawas CH, Keene CD, Kouri N, Kovacs GG, Leverenz JB, Latimer CS, Mackenzie IR, Mao Q, McAleese KE, Merrick R, Montine TJ, Murray ME, Myllykangas L, Nag S, Neltner JH, Newell KL, Rissman RA, Saito Y, Sajjadi SA, Schwetye KE, Teich AF, Thal DR, Tomé SO, Troncoso JC, Wang SJ, White CL 3rd, Wisniewski T, Yang HS, Schneider JA, Dickson DW, Neumann M (2023) LATE-NC staging in routine neuropathologic diagnosis: an update. Acta Neuropathol 145:159–173. 10.1007/s00401-022-02524-2 36512061 10.1007/s00401-022-02524-2 PMC9849315

[45] Pham CT, de Silva R, Haïk S, Verny M, Sachet A, Forette B, Lees A, Hauw JJ, Duyckaerts C (2011) Tau-positive grains are constant in centenarians’ hippocampus. Neurobiol Aging 32:1296–1303. 10.1016/j.neurobiolaging.2009.07.009 19695742 10.1016/j.neurobiolaging.2009.07.009

[46] Purohit DP, Perl DP, Haroutunian V, Powchik P, Davidson M, Davis KL (1998) Alzheimer disease and related neurodegenerative diseases in elderly patients with schizophrenia: a postmortem neuropathologic study of 100 cases. Arch Gen Psychiatry 55:205–211. 10.1001/archpsyc.55.3.205 9510214 10.1001/archpsyc.55.3.205

[47] Ramos-Campoy O, Ávila-Polo R, Grau-Rivera O, Antonell A, Clarimón J, Rojas-García R, Charif S, Santiago-Valera V, Hernandez I, Aguilar M, Almenar C, Lopez-Villegas D, Bajo L, Pastor P, Van der Zee J, Lladó A, Sanchez-Valle R, Gelpi E (2018) Systematic screening of ubiquitin/p62 aggregates in cerebellar cortex expands the neuropathological phenotype of the* C9orf72* expansion mutation. J Neuropathol Exp Neurol 77:703–709. 10.1093/jnen/nly047 29889265 10.1093/jnen/nly047

[48] Rodriguez RD, Suemoto CK, Molina M, Nascimento CF, Leite RE, de Lucena Ferretti-Rebustini RE, Farfel JM, Heinsen H, Nitrini R, Ueda K, Pasqualucci CA, Jacob-Filho W, Yaffe K, Grinberg LT (2016) Argyrophilic grain disease: demographics, clinical, and neuropathological features from a large autopsy study. J Neuropathol Exp Neurol 75:628–635. 10.1093/jnen/nlw034 27283329 10.1093/jnen/nlw034 PMC4913431

[49] Roemer SF, Grinberg LT, Crary JF, Seeley WW, McKee AC, Kovacs GG, Beach TG, Duyckaerts C, Ferrer IA, Gelpi E, Lee EB, Revesz T, White CL 3rd, Yoshida M, Pereira FL, Whitney K, Ghayal NB, Dickson DW (2022) Rainwater Charitable Foundation criteria for the neuropathologic diagnosis of progressive supranuclear palsy. Acta Neuropathol 144:603–614 35947184 10.1007/s00401-022-02479-4 PMC9468104

[50] Sabbagh MN, Sandhu SS, Farlow MR, Vedders L, Shill HA, Caviness JN, Connor DJ, Sue L, Adler CH, Beach TG (2009) Correlation of clinical features with argyrophilic grains at autopsy. Alzheimer Dis Assoc Disord 23:229–233. 10.1097/WAD.0b013e318199d833 19812464 10.1097/WAD.0b013e318199d833 PMC2760041

[51] Saito Y, Nakahara K, Yamanouchi H, Murayama S (2002) Severe involvement of ambient gyrus in dementia with grains. J Neuropathol Exp Neurol 61:789–796. 10.1093/jnen/61.9.789 12230325 10.1093/jnen/61.9.789

[52] Saito Y, Yamazaki M, Kanazawa I, Murayama S (2002) Severe involvement of the ambient gyrus in a case of dementia with argyrophilic grain disease. J Neurol Sci 196:71–75. 10.1016/s0022-510x(02)00027-8 11959159 10.1016/s0022-510x(02)00027-8

[53] Saito Y, Ruberu NN, Sawabe M, Arai T, Tanaka N, Kakuta Y, Yamanouchi H, Murayama S (2004) Staging of argyrophilic grains: an age-associated tauopathy. J Neuropathol Exp Neurol 63:911–918. 10.1093/jnen/63.9.911 15453090 10.1093/jnen/63.9.911

[54] Sakae N, Santos OA, Pedraza O, Litvan I, Murray ME, Duara R, Uitti RJ, Wszolek ZK, Graff-Radford NR, Josephs KA, Dickson DW (2020) Clinical and pathologic features of cognitive-predominant corticobasal degeneration. Neurology 95:e35–e45. 10.1212/WNL.0000000000009734 32518146 10.1212/WNL.0000000000009734 PMC7371382

[55] Sakurai K, Tokumaru AM, Ikeda T, Morimoto S, Inui S, Sumida K, Oba H, Nakagawa M, Matsukawa N, Hashizume Y (2019) Characteristic asymmetric limbic and anterior temporal atrophy in demented patients with pathologically confirmed argyrophilic grain disease. Neuroradiology 61:1239–1249. 10.1007/s00234-019-02247-4 31256221 10.1007/s00234-019-02247-4

[56] Sakurai K, Kaneda D, Morimoto S, Uchida Y, Inui S, Kimura Y, Kato T, Ito K, Hashizume Y (2022) Clinicoradiological features in progressive supranuclear palsy comorbid with argyrophilic grains. Mov Disord Clin Pract 9:484–488. 10.1002/mdc3.13455 35586531 10.1002/mdc3.13455 PMC9092728

[57] Santpere G, Ferrer I (2009) Delineation of early changes in cases with progressive supranuclear palsy-like pathology. Astrocytes in striatum are primary targets of tau phosphorylation and GFAP oxidation. Brain Pathol 19:177–187. 10.1111/j.1750-3639.2008.00173.x 18462470 10.1111/j.1750-3639.2008.00173.x PMC8094872

[58] Shi Y, Zhang W, Yang Y, Murzin AG, Falcon B, Kotecha A, van Beers M, Tarutani A, Kametani F, Garringer HJ, Vidal R, Hallinan GI, Lashley T, Saito Y, Murayama S, Yoshida M, Tanaka H, Kakita A, Ikeuchi T, Robinson AC, Mann DMA, Kovacs GG, Revesz T, Ghetti B, Hasegawa M, Goedert M, Scheres SHW (2021) Structure-based classification of tauopathies. Nature 598:359–363. 10.1038/s41586-021-03911-7 34588692 10.1038/s41586-021-03911-7 PMC7611841

[59] Taniguchi-Watanabe S, Arai T, Kametani F, Nonaka T, Masuda-Suzukake M, Tarutani A, Murayama S, Saito Y, Arima K, Yoshida M, Akiyama H, Robinson A, Mann DMA, Iwatsubo T, Hasegawa M (2016) Biochemical classification of tauopathies by immunoblot, protein sequence and mass spectrometric analyses of sarkosyl-insoluble and trypsin-resistant tau. Acta Neuropathol 131:267–280. 10.1007/s00401-015-1503-3 26538150 10.1007/s00401-015-1503-3 PMC4713716

[60] Tarutani A, Adachi T, Akatsu H, Hashizume Y, Hasegawa K, Saito Y, Robinson AC, Mann DMA, Yoshida M, Murayama S, Hasegawa M (2022) Ultrastructural and biochemical classification of pathogenic tau, α-synuclein and TDP-43. Acta Neuropathol 143:613–640. 10.1007/s00401-022-02426-3 35513543 10.1007/s00401-022-02426-3 PMC9107452

[61] Thal DR, Rüb U, Orantes M, Braak H (2002) Phases of Aβ-deposition in the human brain and its relevance for the development of AD. Neurology 58:1791–1800. 10.1212/wnl.58.12.1791 12084879 10.1212/wnl.58.12.1791

[62] Tsuchiya K, Mitani K, Arai T, Yamada S, Komiya T, Esaki Y, Haga C, Yamanouchi H, Ikeda K (2001) Argyrophilic grain disease mimicking temporal Pick’s disease: a clinical, radiological, and pathological study of an autopsy case with a clinical course of 15 years. Acta Neuropathol 102:195–199. 10.1007/s004010100365 11563637 10.1007/s004010100365

[63] Uchikado H, Lin WL, DeLucia MW, Dickson DW (2006) Alzheimer disease with amygdala Lewy bodies: a distinct form of alpha-synucleinopathy. J Neuropathol Exp Neurol 65:685–697. 10.1097/01.jnen.0000225908.90052.07 16825955 10.1097/01.jnen.0000225908.90052.07 PMC5706655

[64] Yokota O, Tsuchiya K, Terada S, Ishizu H, Uchikado H, Ikeda M, Oyanagi K, Nakano I, Murayama S, Kuroda S, Akiyama H (2008) Basophilic inclusion body disease and neuronal intermediate filament inclusion disease: a comparative clinicopathological study. Acta Neuropathol 115:561–575. 10.1007/s00401-007-0329-z 18080129 10.1007/s00401-007-0329-z

[65] Yokota O, Tsuchiya K, Arai T, Yagishita S, Matsubara O, Mochizuki A, Tamaoka A, Kawamura M, Yoshida H, Terada S, Ishizu H, Kuroda S, Akiyama H (2009) Clinicopathological characterization of Pick’s disease versus frontotemporal lobar degeneration with ubiquitin/TDP-43-positive inclusions. Acta Neuropathol 117:429–444. 10.1007/s00401-009-0493-4 19194716 10.1007/s00401-009-0493-4

[66] Yokota O, Miki T, Haraguchi T, Terada S, Yamada N (2022) Argyrophilic grain disease: clinical and pathological characteristics. Dementia Jpn 36:53–66 (**Japanese with English abstract**)

[67] Yokota O, Miki T, Ikeda C, Ishizu H, Haraguchi T, Miyashita A, Ikeuchi T, Takenoshita S, Terada S (2022) Amygdala granular fuzzy astrocytes as lesions preceding development of argyrophilic grains: data from 239 autopsy cases. Free Neuropathol 3:3–18. 10.17879/freeneuropathology-2022-4285 37284152 10.17879/freeneuropathology-2022-4285 PMC10240936

[68] Yokota O, Miki T, Nakashima-Yasuda H, Ishizu H, Haraguchi T, Ikeda C, Miyashita A, Ikeuchi T, Takenoshita S, Terada S, Takaki M (2023) Amygdala granular fuzzy astrocytes are independently associated with both LATE neuropathologic change and argyrophilic grains: a study of Japanese series with a low to moderate Braak stage. Acta Neuropathol Commun 11:148. 10.1186/s40478-023-01643-5 37697414 10.1186/s40478-023-01643-5 PMC10496338

[69] Yoshida K, Hata Y, Ichimata S, Okada K, Nishida N (2023) Argyrophilic grain disease is common in older adults and may be a risk factor for suicide: a study of Japanese forensic autopsy cases. Transl Neurodegener 12:16. 10.1186/s40035-023-00352-2 37004112 10.1186/s40035-023-00352-2 PMC10067165

