# Supplementary figures and images for: Pure argyrophilic grain disease revisited: independent effects on limbic, neocortical, and striato-pallido-nigral degeneration and the development of dementia in a series with a low to moderate Braak stage
Source: Acta Neuropathol Commun. 2024 Jul 31;12:121. doi: 10.1186/s40478-024-01828-6 (PMC11290173; doi:10.1186/s40478-024-01828-6)

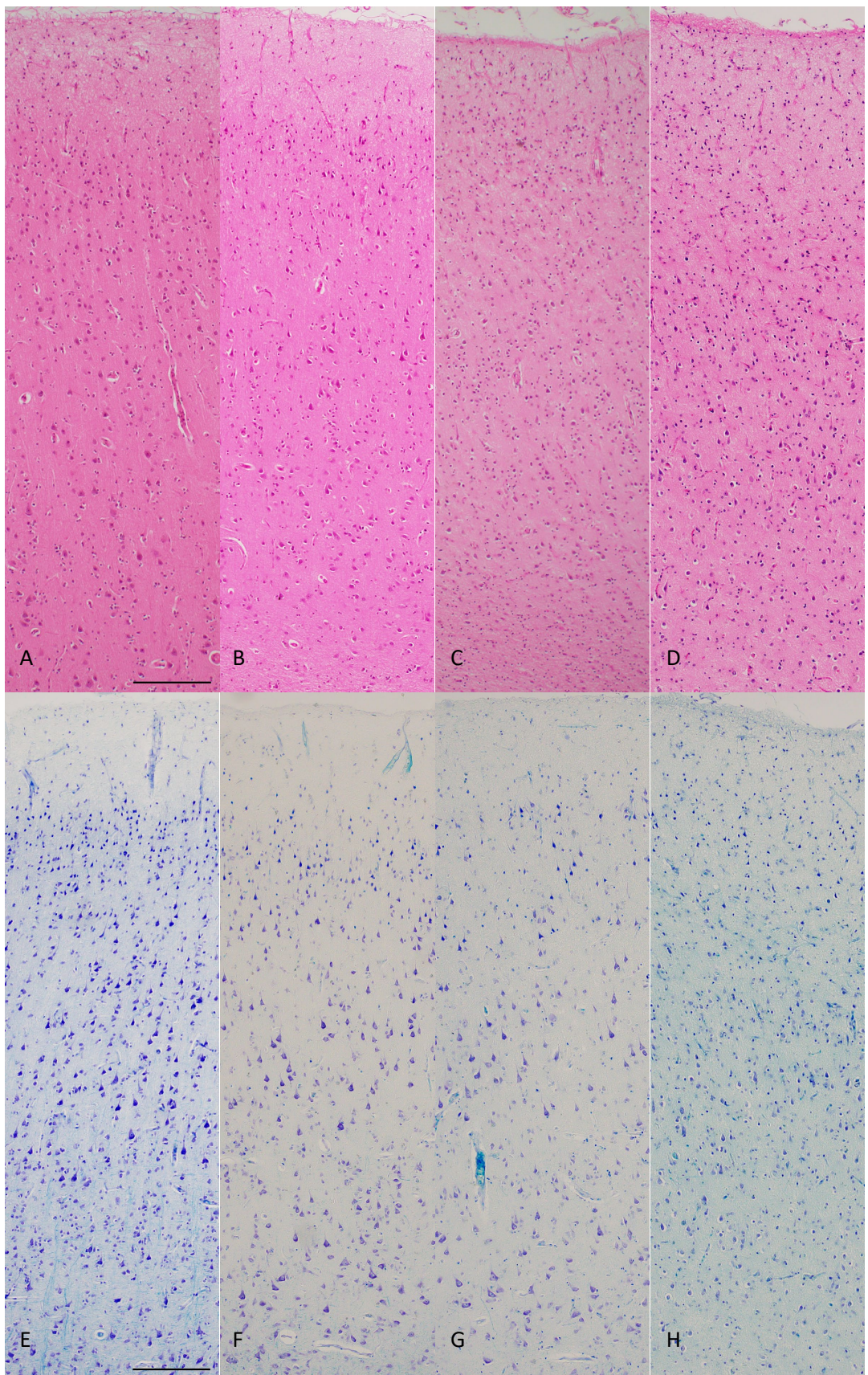

Supplementary figure 1

Supplement: Supplementary file 1 — Supplementary figure 1. Representative photos of the grading system (stages 0–4) of neuronal loss with gliosis in the cerebral cortex. A, E Stage 0. No neuronal loss. B, F Stage 1. Mild neuronal loss. C, G Stage 2. Moderate neuronal loss. D, H Stage 3. Severe neuronal loss. A–D H&E stain, E–H KB stain. Scale bars: A–D 200 μm, E–H 200 μm. The most severe grade was recorded as the stage in each region. Details of the definition of each stage are noted in the text. [file 40478_2024_1828_MOESM1_ESM.pdf]

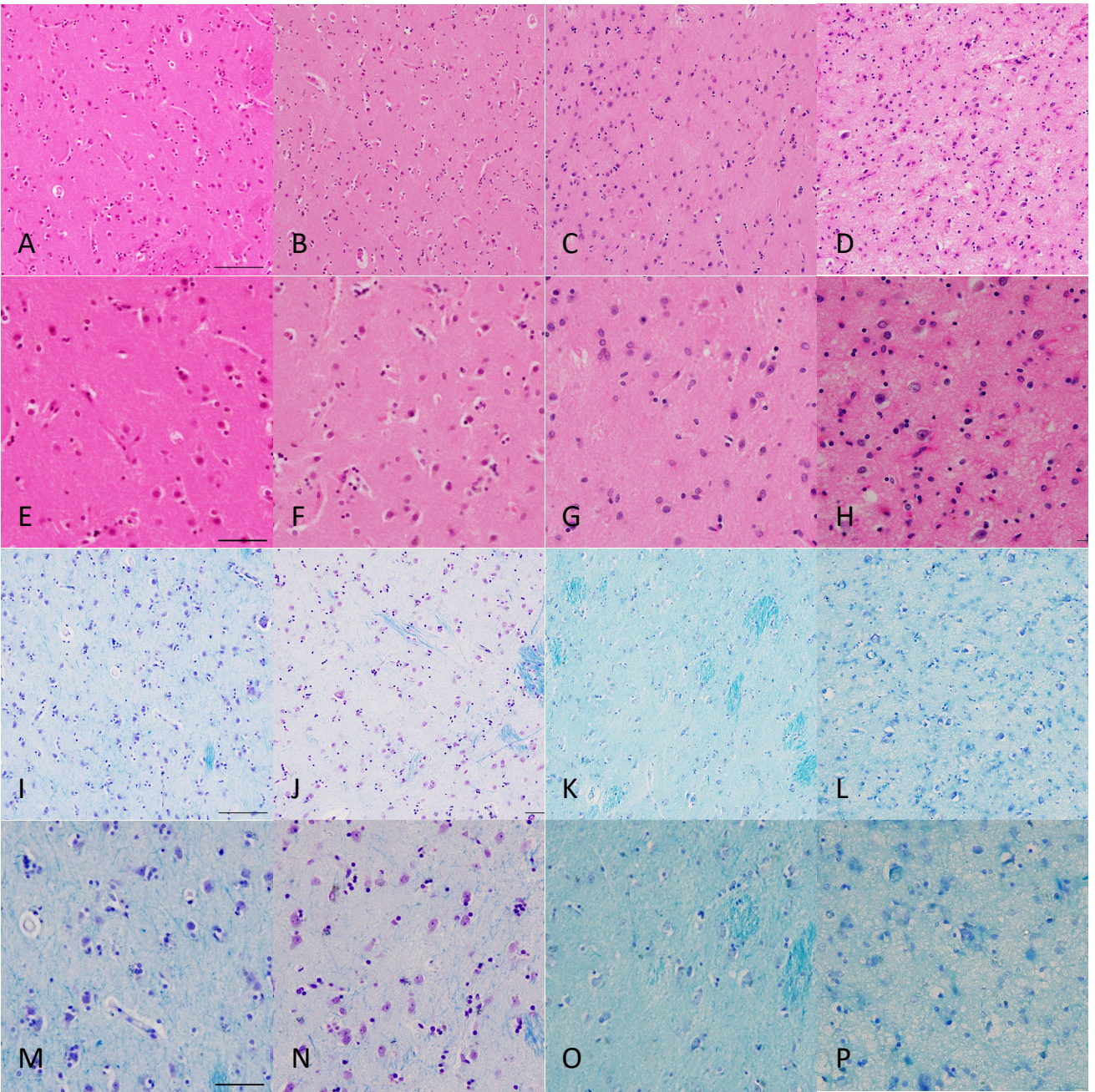

Supplementary figure 2

Supplement: Supplementary file 2 — Supplementary figure 2. Representative photos of the grading system (stages 0–4) of neuronal loss with gliosis in the subcortical nuclei. A, E, I, M Stage 0. No neuronal loss. B, F, J, N Stage 1. Mild neuronal loss. C, G, K, O Stage 2. Moderate neuronal loss. D, H, L, P Stage 3. Severe neuronal loss. A–H H&E stain, I–P KB stain. Scale bars: A–D, I–L 100 μm, E–H, M–P 50 μm. The most severe grade was recorded as the stage in each region. Details of the definition of each stage are noted in the text. [file 40478_2024_1828_MOESM2_ESM.pdf]

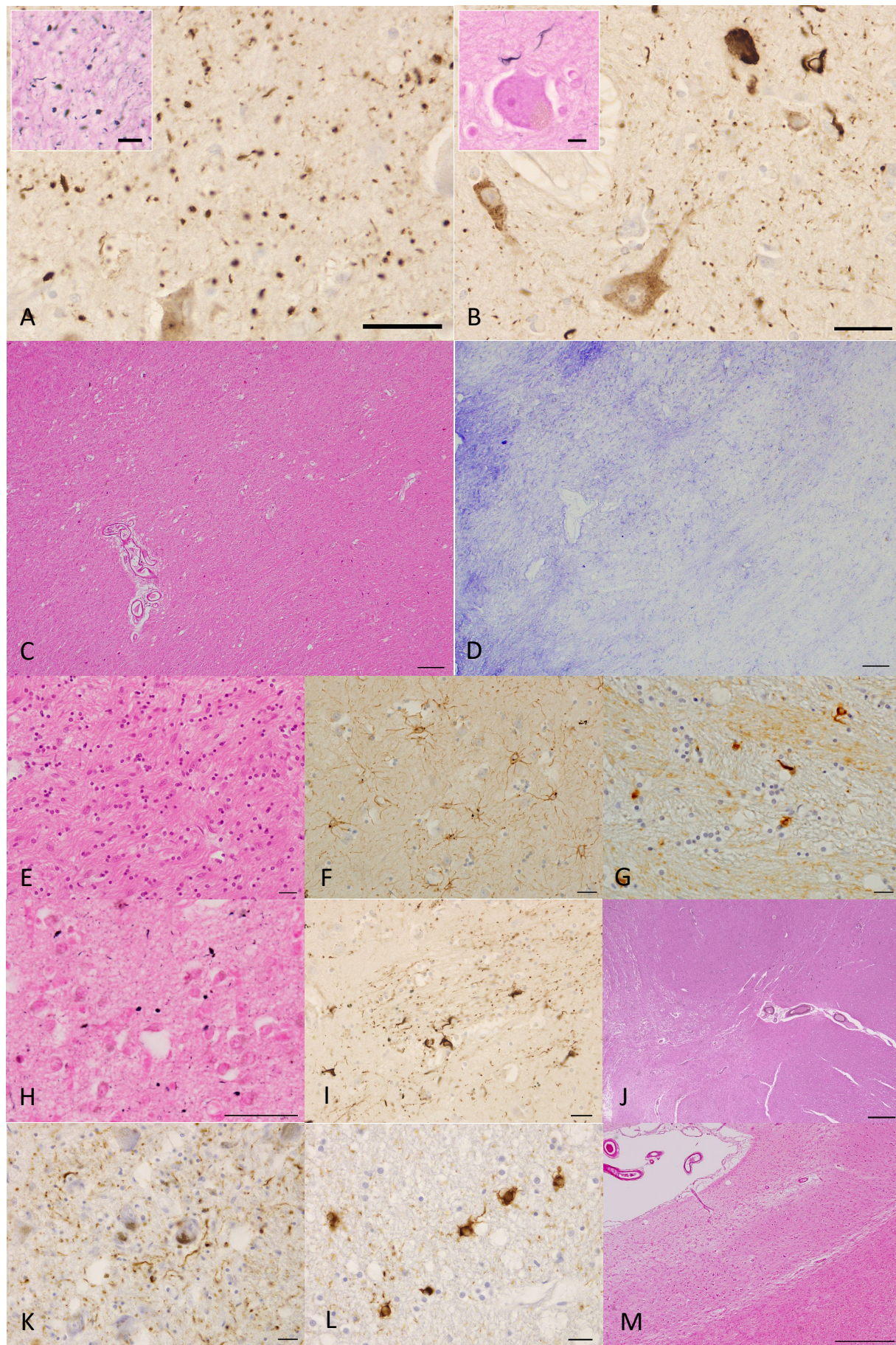

Supplementary figure 3

Supplement: Supplementary file 3 — Supplementary figure 3. Pathological findings in case 3. A 4R tau-positive AGs in the amygdala. Scale bar = 30 μm. Inset: Argyrophilic grains in the same region. Gallyas method. Scale bar = 10 μm. B 4R tau-positive Betz cell and argyrophillic grains in the primary motor cortex. Scale bar = 30 μm. Inset: The Gallyas method did not demonstrate argyrophilia in Betz cells. Scale bar = 10 μm. C Severe neuronal loss with gliosis in the globus pallidus. Tissue degeneration is more evident in the dorsal portion of the site. H&E stain. Scale bar = 200 μm. D Holzer stain demonstrated severe gliosis in the dorsal portion rather than the ventral portion in the globus pallidus. Scale bar = 200 μm. E, F Severe neuronal loss and gliosis in the globus pallidus demonstrated by H&E stain (E) and GFAP immunohistochemistry (F). Scale bar = 20 μm. G AT8-positive neurons and threads in the globus pallidus. Scale bar = 20 μm. H AGs in the caudate nucleus. Gallyas method. Scale bar = 50 μm. I AT8-positive NFTs and threads in the putamen. Scale bar = 20 μm. J Severe neuronal loss in the substantia nigra. H&E stain. Scale bar = 600 μm. K AT8-positive NFTs and threads in the substantia nigra. Scale bar = 20 μm. L AT8-positive glial cells and fine threads in the white matter in the cerebellum. Scale bar = 20 μm. M Hippocampal sclerosis characterized by severe loss in the CA1 and subiculum. H&E stain. Scale bar = 500 μm. [file 40478_2024_1828_MOESM3_ESM.pdf]
