# Supplementary material for: Pure argyrophilic grain disease revisited: independent effects on limbic, neocortical, and striato-pallido-nigral degeneration and the development of dementia in a series with a low to moderate Braak stage
Source: Acta Neuropathol Commun. 2024 Jul 31;12:121. doi: 10.1186/s40478-024-01828-6 (PMC11290173; doi:10.1186/s40478-024-01828-6)
Supplement: Supplementary file 4 — Supplementary file 1 [file 40478_2024_1828_MOESM4_ESM.docx]

**Supplementary file 1**

**Clinical courses of diffuse form of pAGD cases**

***Case 1***

This patient was a right-handed unmarried Japanese woman who was 86 years old at the time of death. Her hobby was collecting tableware for tea service. From the age of 49 years, she began to purchase very expensive items frequently and generously give the items to her neighbors, and her relatives began to find it strange. At age 67, stereotypic behaviors (e.g., she repeatedly picked up cigarette butts on the street and placed them on the opposite side of the street) also gradually developed. Relatives noticed she had memory impairment by age 74, and she was first examined in our memory clinic at a psychiatric hospital. She had no family or past history of neurological or psychiatric disorders. Neurological examination did not demonstrate impairment of eye movement, dysarthria, dysphagia, or parkinsonism. Blood examinations were normal. The score of the Hasegawa dementia scale revised (HDS-R), which is most frequently employed to assess cognitive function of people with dementia in Japan and correlates well with the score of the Mini Mental State Examination (MMSE), was 18/30 points (cutoff 19/20). Her daily living activity was independent. A head computed tomography (CT) scan showed atrophy in the amygdala and hippocampus. The neocortex was relatively well spared (**Figures 1A**). She was diagnosed with Alzheimer’s disease. At age 75, she was admitted to a psychiatric hospital. Her attitude was polite but showed hypermetamorphosis. At age 76, indifference became evident and her appetite increased. She scored 28/100 on Addenbrooke’s cognitive examination (cut off: 82/83, VLOM ratio 3.6) and 17/30 on the MMSE (cut off: 24/25). Naming performance was moderately impaired, and semantic memory impairment on common objects was noted: for example, she correctly named a clock, pencil, and pig, but she called a camel a ‘horse,’ and a windmill a ‘building’, and she said ‘What’s this? I don’t know what that is’ when she saw pictures of a giraffe and kangaroo. At age 76, she scored 8/18 points on the Frontal Assessment Battery. From the age 78, forced behaviors induced by visual stimuli and behavioral stereotypy became remarkable. However, she could cooperate in a neurological examination by following instructions (e.g., open her mouth, put out her tongue, raise her right or left hand, turn her face upward) until the age 79. At age 84, speech output gradually decreased. Dysphagia and rigidity in the neck and in the bilateral upper extremities with right side-predominance developed. Impairment of eye movement, tremor, pyramidal sign, muscle atrophy, and hemineglect were not noted, and she could walk with support. CT images at age 86 showed severe slightly left side-predominant atrophy in the amygdala, hippocampus, and temporal cortex, and symmetric moderate diffuse atrophy in the frontal and parietal cortices. The left temporal tip showed knife-edge atrophy (**Figures 1B, 1C**). She died of pneumonia after a disease duration of 39 years.

***Case 2***

Relatives of the patient, a right-handed Japanese man, first noticed that he could not recognize their faces at the age of 64 years. At age 68, he was first examined at the outpatient neurology department at a general hospital. He had no family or past history of neurological or psychiatric disorders. Magnetic resonance imaging (MRI) showed right side-predominant atrophy in the amygdala. The temporal lobes including the tips also showed right side-predominant atrophy (**Figures 3A, 3B**). He scored 25/30 points on the MMSE. He was diagnosed with AD. At age 70, stereotypic behaviors gradually occurred: he repeatedly went to a store and excessively bought notebooks, handicraft materials, and sweet breads. He burned fallen leaves at a shrine and repeatedly tidied up every day, and didn't stop even when the police warned him. At age 71, he was examined at a memory clinic in a psychiatric hospital. He showed remarkable impairment of recognition of famous persons’ faces: in the task of recognizing photos of famous persons who he definitely should know and naming them, he consistently answered ‘I don’t know the person’. However, he could spontaneously provide their superordinate category (e.g., an actor or athlete, etc.). In contrast to the severe impairment of the recognition of persons, semantic memory of common objects was well spared. His appetite was increased, and he tended to eat bread excessively. He appeared to walk normally, but his wife noticed that he gradually became slower. Mild rigidity was noted in the left leg, while muscle tone was normal in the other extremities and neck. Impairment of eye movement, tremor, muscle atrophy, weakness, fasciculation, Babinski sign, and ankle clonus were absent. The MMSE score was 22/30, and HDS-R score was 21/30. Single photon emission computed tomography (SPECT) revealed hypoperfusion in the bilateral amygdala (**Figure 3E, 3F**). He was diagnosed with semantic dementia (recognition impairment of persons). At age 72, his stereotypic behaviors were worsened and sexual disinhibition also occurred. Rigidity in the left upper and lower extremities became evident. At age 74, MRI demonstrated the progression of atrophy in the limbic region and temporo-frontal cortex (**Figures 3C, 3D**). At age 75, dysphagia and falls occurred. At age 79, he could not walk without support. At age 80, left side-predominant rigidity in the bilateral extremities occurred. Impairment of eye movement, Babinski sign, ankle clonus, muscle atrophy, fasciculation, semantic memory impairment of common objects, and hemineglect were not noted. He was still able to have simple conversations, and communication was bidirectional. For example, when asked to write a sentence on a piece of paper, he jokingly wrote, "I want to be a very attractive guy." He died of pneumonia at age 81 with the disease duration of 17 years.

***Case 3***

The patient was a Japanese man who was 60 years old at the time of death. He had no family history of neurological or psychiatric disorders. Clinical data on this patient, who died in the mid-1990s, were limited. He first developed behavioral change at age 40. He started trying to take people's things, and he was arrested by the police after breaking into a neighbor's home without permission. He was noticed repeating the same behaviors. At age 55, he was admitted to a psychiatric hospital. Disorientation, euphoria, and talkativeness were noted. Memory impairment was not noted. On the Wechsler Adult Intelligence Scale-Revised (WAIS-R), he obtained a verbal IQ score of 72 and performance IQ score of 78. At age 57, oral tendency, indifference, and double incontinence were noted. At age 58, he became bedridden. He died of gastric bleeding 20 years after the onset.
