# Supplementary material for: Pure argyrophilic grain disease revisited: independent effects on limbic, neocortical, and striato-pallido-nigral degeneration and the development of dementia in a series with a low to moderate Braak stage
Source: Acta Neuropathol Commun. 2024 Jul 31;12:121. doi: 10.1186/s40478-024-01828-6 (PMC11290173; doi:10.1186/s40478-024-01828-6)
Supplement: Supplementary file 5 — Supplementary tables [file 40478_2024_1828_MOESM5_ESM.pdf]

**Supplementary table 1. Antibodies used in this study**

| Antibody | Species/type | Dilution | Epitope | Source |
|---|---|---|---|---|
| AT8 | Mouse/monoclonal | 1:1,000 | Tau phosphorylated at Ser 202 | Innogenetics |
| RD3 | Mouse/monoclonal | 1:2,000 | 3-repeat tau-specific anti-tau antibody | Merck Millipore |
| RD4 | Mouse/monoclonal | 1:100 | 4-repeat tau-specific anti-tau antibody | Merck Millipore |
| Anti-4R tau | Rabbit/polyclonal | 1:2,000 | 4-repeat tau-specific anti-tau antibody | Cosmo Bio |
| 12B2 | Mouse/monoclonal | 1:100 | Aβ(11–28) | IBL |
| psyn#64 | Mouse/monoclonal | 1:5,000 | Phosphorylated α-synuclein | Wako |
| pS409/410-2 | Rabbit/polyclonal | 1:5,000 | Phosphorylated TDP-43 | Cosmo Bio |
| HPA008784 | Rabbit/polyclonal | 1:200 | FUS | Sigma-Aldrich |
| SMI31 | Mouse/monoclonal | 1:1,000 | Phosphorylated neurofilament | Sternberger |
| p62-N | Guinea pig/polyclonal | 1:100 | N-terminus of p62 protein | Progen Biotechnik |
| p62-C | Guinea pig/polyclonal | 1:500 | C-terminus of p62 protein | Progen Biotechnik |

## Supplementary table 2. Clinical and pathological features in diffuse form pAGD cases

| | Case 1 | | | | Case 2 | | | | Case 3 | | | |
|---|---|---|---|---|---|---|---|---|---|---|---|---|
| ***Clinical features*** | | | | | | | | | | | | |
| Age at onset (y) | 49 | | | | 64 | | | | 44 | | | |
| Age at death (y) | 86 | | | | 81 | | | | 60 | | | |
| Disease duration (y) | 38 | | | | 17 | | | | 16 | | | |
| Sex | Female | | | | Male | | | | Male | | | |
| Family history | - | | | | - | | | | - | | | |
| MAPT mutation | - | | | | - | | | | n.a. | | | |
| Apo E genotype | ε3/ε3 | | | | ε3/ε3 | | | | n.a. | | | |
| Clinical diagnosis | bvFTD | | | | Right temporal variant FTD | | | | Manic state | | | |
| Onset symptoms | Lavish expenditure | | | | Impairment of face recognition | | | | Breaking into a residence, behavioral stereotypy | | | |
| Behavioral stereotypy | + | | | | + | | | | + | | | |
| Semantic memory impairment | + | | | | - | | | | n.a. | | | |
| Prosopagnosia | - | | | | + | | | | n.a. | | | |
| Impairment of eye movement | - | | | | - | | | | n.a. | | | |
| Parkinsonism | + (R>L) | | | | + (L>R) | | | | n.a. | | | |
| Pyramidal sign | - | | | | - | | | | n.a. | | | |
| Lower motor neuron sign | - | | | | - | | | | n.a. | | | |
| | | | | | | | | | | | | |
| ***Pathological features*** | | | | | | | | | | | | |
| Brain weight (g) | 856 | | | | 1,188 | | | | 1,040 | | | |
| Post-mortem interval (h) | 3.0 | | | | 2.5 | | | | 2.1 | | | |
| Braak NFT stage | Stage II | | | | Stage IV | | | | Stage I | | | |
| Thal phase | Phase 0 | | | | Phase 1 | | | | Phase 0 | | | |
| CERAD neuritic plaque score | 0 | | | | 0 | | | | 0 | | | |
| Lewy body disease | - | | | | - | | | | - | | | |
| Braak Parkinson's disease | Stage 0 | | | | Stage 0 | | | | Stage 0 | | | |
| LATE-NC stage/Josephs stage | Stage 3/6 | | | | Stage 2/3 | | | | Stage 0/0 | | | |
| TDP-43 histologic subtype | Type β | | | | Type β | | | | - | | | |
| FUS pathology | - | | | | - | | | | - | | | |
| Saito AG stage | Stage III (diffuse form) | | | | Stage III (diffuse form) | | | | Stage III (diffuse form) | | | |
| | | | | | | | | | | | | |
| Lesions | NL | AG | NFT | GFA | NL | AG | NFT | GFA | NL | AG | NFT | GFA |
| Motor cortex | + | 0.5 | ++ | +++ | - | 0.5 | ++ | +++ | - | 0.5 | ++ | +++ |
| Middle frontal gyrus | + | + | ++ | +++ | ++ | 0.5 | ++ | +++ | ++ | 0.5 | +++ | +++ |
| Orbital gyrus | + | ++ | ++ | +++ | + | 0.5 | ++ | +++ | ++ | ++ | +++ | + |
| Superior temporal gyrus | + | ++ | +++ | +++ | + | + | ++++ | +++ | + | ++ | + | - |
| Inferior temporal gyrus | +++ | + | ++ | ++ | ++ | ++ | ++ | +++ | ++ | +++ | +++ | - |
| Inferior parietal lobule | + | 0.5 | ++ | ++ | + | 0.5 | ++ | +++ | + | 0.5 | ++ | - |
| Striate region | + | - | - | - | - | - | - | + | - | - | - | - |
| Insular cortex | +++ | +++ | ++ | +++ | +++ | ++ | ++ | +++ | +++ | ++ | +++ | + |
| Caudate nucleus | +++ | 0.5 | ++++ | +++ | ++ | 0.5 | ++ | +++ | +++ | 0.5 | ++++ | - |
| Putamen | ++ | 0.5 | +++ | +++ | + | 0.5 | +++ | +++ | ++ | 0.5 | ++ | - |
| Globus pallidus, internal segment | +++ | - | ++ | ++ | +++ | - | ++ | ++ | +++ | - | + | - |
| Globus pallidus, external segment | ++ | - | +++ | ++ | ++ | - | ++ | +++ | +++ | - | + | - |
| Thalamus | ++ | 0.5 | ++ | - | ++ | 0.5 | ++ | ++ | +++ | 0.5 | ++ | - |
| Subthalamic nucleus | - | - | ++ | - | - | 0.5 | ++ | + | - | 0.5 | ++ | - |
| Amygdala | +++ | +++ | +++ | +++ | +++ | ++ | ++++ | +++ | +++ | +++ | ++ | ++ |
| Ambient gyrus | +++ | +++ | ++ | +++ | +++ | ++ | +++++ | +++ | +++ | ++ | ++ | ++ |
| Hippocampal CA1 | +++ | ++ | ++ | - | +++ | ++ | ++++ | - | +++ | +++ | +++ | - |
| Hippocampal dentate gyrus | - | - | +++ | - | - | + | +++++ | - | - | - | ++ | - |
| Parahippocampal gyrus | +++ | 0.5 | +++ | +++ | +++ | 0.5 | ++++ | +++ | +++ | + | ++ | - |
| Oculomotor nucleus | - | - | ++ | + | + | 0.5 | ++ | +++ | - | 0.5 | + | + |
| Red nucleus | - | 0.5 | ++ | - | - | 0.5 | ++ | +++ | - | 0.5 | ++ | ++ |
| Substantia nigra | +++ | - | +++ | - | +++ | 0.5 | ++ | + | +++ | 0.5 | ++ | + |
| Locus coeruleus | - | 0.5 | +++ | - | ++ | 0.5 | ++ | - | ++ | 0.5 | ++ | - |
| Superior cerebellar peduncle | - | - | NT+ | - | - | - | NT+ | - | - | - | NT++ | - |
| Pontine nucleus | - | - | ++ | + | - | 0.5 | ++ | + | - | - | ++ | + |
| Hypoglossal nucleus | - | 0.5 | - | + | - | 0.5 | - | - | - | - | - | - |
| Dorsal vagal nucleus | - | 0.5 | ++ | - | - | - | ++ | - | - | 0.5 | ++ | + |
| Inferior olivary nucleus | + | 0.5 | ++ | + | - | - | + | ++ | - | 0.5 | + | ++ |
| Frontopontine tract | + | - | NT++ | - | - | - | NT+ | - | +++ | 0.5 | NT++ | - |
| Corticospinal tract (cerebral peduncle) | - | - | NT+ | - | - | - | - | - | - | - | NT+ | - |
| Corticospinal tract (medulla oblongata) | - | - | NT+ | - | - | - | NT+ | - | - | - | NT+ | - |
| Corticospinal tract (cervical cord) | - | - | - | - | - | - | - | - | n.a. | n.a. | n.a. | n.a. |
| Anterior horn cells | - | - | + | + | - | - | NT+ | + | n.a. | n.a. | n.a. | n.a. |
| Cerebellar cortex | - | - | - | - | - | - | - | - | - | - | - | - |
| Dentate nucleus | - | - | + | - | - | - | ++ | + | - | - | + | - |

y: years, MAPT: microtubule associated protein tau, bvFTD: behavioral variant frontotemporal dementia, +: present, -: absent, R>L: right side predominant, L>R: left side predominant, n.a. not

available, h: hours, CERAD: the Consortium to Establish a Registry for Alzheimer's Disease, LATE-NC: limbic-predominant age-related TDP-43 encephalopathy neuropathologic changes, TDP-43: TAR DNA-binding protein of 43 kDa, FUS: fused in sarcoma, AGD: argyrophilic grain disease, NL: neuronal loss, AG: argyrophilic grains, NFT: neurofibrillary tangles and threads, GFA: granular fuzzy astrocytes. The grading system of each lesion is noted in the text.

**Supplementary table 3. Correlations between the age at death, Braak NFT stage, Thal phase, Saito AG stage, GFA stage, LATE-NC stage and severity of neuronal loss in pAGD cases**

| | Age at death | | Braak NFT stage | | Thal phase | | Saito AG stage | | GFA stage (frontal cortex) | | GFA stage (caudate nucleus) | | GFA stage (putamen) | | GFA stage (amygdala) | | LATE-NC stage | |
|---|---|---|---|---|---|---|---|---|---|---|---|---|---|---|---|---|---|---|
| | ρ | p | ρ | p | ρ | p | ρ | p | ρ | p | ρ | p | ρ | p | ρ | p | ρ | p |
| Age at death | 1.0000 | - | 0.5469 | 0.0018** | 0.0437 | 0.8188 | 0.3538 | 0.0551 | 0.1504 | 0.4362 | 0.1477 | 0.4360 | 0.1259 | 0.5075 | 0.2160 | 0.2892 | 0.4751 | 0.0080** |
| Braak NFT stage | 0.5469 | 0.0018** | 1.0000 | - | 0.2409 | 0.1997 | 0.1520 | 0.4225 | 0.2105 | 0.2730 | 0.2501 | 0.1826 | 0.3698 | 0.0443* | 0.2019 | 0.3227 | 0.1925 | 0.3082 |
| Thal phase | 0.0437 | 0.8188 | 0.2409 | 0.1997 | 1.0000 | - | 0.2310 | 0.2194 | -0.0911 | 0.6384 | 0.2436 | 0.1946 | 0.0960 | 0.6139 | 0.2544 | 0.2098 | 0.2097 | 0.2661 |
| Saito AG stage | 0.3538 | 0.0551 | 0.1520 | 0.4225 | 0.2310 | 0.2194 | 1.0000 | - | 0.3346 | 0.0760 | 0.2360 | 0.2092 | 0.2825 | 0.1304 | 0.0214 | 0.9172 | 0.4009 | 0.0281* |
| GFA stage (frontal cortex) | 0.1504 | 0.4362 | 0.2105 | 0.2730 | -0.0911 | 0.6384 | 0.3346 | 0.0760 | 1.0000 | - | 0.2134 | 0.2664 | 0.1423 | 0.4614 | -0.1648 | 0.4312 | -0.0096 | 0.9607 |
| GFA stage (caudate nucleus) | 0.1477 | 0.4360 | 0.2501 | 0.1826 | 0.2436 | 0.1946 | 0.2360 | 0.2092 | 0.2134 | 0.2664 | 1.0000 | - | 0.5058 | 0.0044** | 0.3801 | 0.0554 | 0.1818 | 0.3363 |
| GFA stage (putamen) | 0.1259 | 0.5075 | 0.3698 | 0.0443* | 0.0960 | 0.6139 | 0.2825 | 0.1304 | 0.1423 | 0.4614 | 0.5058 | 0.0044** | 1.0000 | - | -0.0011 | 0.9956 | 0.1669 | 0.3781 |
| GFA stage (amygdala) | 0.2160 | 0.2892 | 0.2019 | 0.3227 | 0.2544 | 0.2098 | 0.0214 | 0.9172 | -0.1648 | 0.4312 | 0.3801 | 0.0554 | -0.0011 | 0.9956 | 1.0000 | - | 0.1683 | 0.4111 |
| LATE-NC stage | 0.4751 | 0.0080** | 0.1925 | 0.3082 | 0.2097 | 0.2661 | 0.4009 | 0.0281* | -0.0096 | 0.9607 | 0.1818 | 0.3363 | 0.1669 | 0.3781 | 0.1683 | 0.4111 | 1.0000 | - |
| Neuronal loss stage | | | | | | | | | | | | | | | | | | |
| Amygdala | 0.3079 | 0.1182 | 0.0828 | 0.6812 | -0.0636 | 0.7525 | 0.5744 | 0.0017** | 0.4022 | 0.0417* | 0.0352 | 0.8616 | -0.1084 | 0.5903 | -0.2321 | 0.2539 | 0.2505 | 0.2075 |
| Ambient gyrus | 0.3807 | 0.0501 | 0.1493 | 0.4573 | -0.0766 | 0.7040 | 0.6732 | < 0.001** | 0.3022 | 0.1335 | 0.1989 | 0.3198 | 0.1051 | 0.6019 | -0.1319 | 0.5205 | 0.3403 | 0.0824 |
| Entorhinal gyrus | 0.3375 | 0.0852 | 0.1090 | 0.5882 | 0.0623 | 0.7574 | 0.7517 | < 0.001** | 0.3709 | 0.0621 | 0.1001 | 0.6192 | -0.0299 | 0.8823 | -0.2519 | 0.2244 | 0.5380 | 0.0038** |
| Hippocampal CA1 | 0.1889 | 0.3265 | 0.1687 | 0.3817 | 0.0826 | 0.6701 | 0.5904 | < 0.001** | 0.3961 | 0.0369* | 0.0643 | 0.7405 | 0.3209 | 0.0897 | -0.3220 | 0.1165 | 0.5052 | 0.0052** |
| Subiculum | 0.3653 | 0.0514 | 0.3484 | 0.0640 | 0.1629 | 0.3986 | 0.4927 | 0.0066** | 0.2608 | 0.1800 | 0.1303 | 0.5006 | 0.4285 | 0.0204* | -0.1532 | 0.4648 | 0.6051 | < 0.001** |
| Insular cortex | 0.2241 | 0.2339 | -0.0452 | 0.8125 | 0.1092 | 0.5656 | 0.6212 | < 0.001** | 0.2841 | 0.1352 | 0.1434 | 0.4497 | 0.0700 | 0.7132 | -0.2243 | 0.2706 | 0.4718 | 0.0085** |
| Superior temporal gyrus | 0.2117 | 0.2891 | -0.0937 | 0.6420 | 0.1655 | 0.4094 | 0.7628 | < 0.001** | 0.3715 | 0.0617 | 0.2684 | 0.1758 | 0.0254 | 0.9001 | -0.0006 | 0.9978 | 0.3566 | 0.0679 |
| Middle temporal gyrus | 0.3114 | 0.0939 | 0.0446 | 0.8150 | 0.2022 | 0.2838 | 0.7526 | < 0.001** | 0.4045 | 0.0295* | 0.2534 | 0.1766 | 0.1181 | 0.5343 | -0.1475 | 0.4721 | 0.4736 | 0.0082** |
| Inferior temporal gyrus | 0.2317 | 0.2180 | -0.0639 | 0.7373 | 0.0814 | 0.6688 | 0.7292 | < 0.001** | 0.4415 | 0.0165* | 0.3040 | 0.1024 | 0.1566 | 0.4084 | -0.0615 | 0.7652 | 0.3853 | 0.0355* |
| Occipitotemporal gyrus | 0.2779 | 0.1370 | -0.0030 | 0.9876 | 0.1062 | 0.5766 | 0.6689 | < 0.001** | 0.3979 | 0.0325* | 0.2095 | 0.2666 | 0.0582 | 0.7600 | -0.2198 | 0.2805 | 0.4406 | 0.0148* |
| Superior frontal gyrus | 0.1933 | 0.3060 | -0.0135 | 0.9436 | 0.2800 | 0.1339 | 0.6284 | < 0.001** | 0.2529 | 0.1857 | 0.1470 | 0.4383 | -0.0445 | 0.8152 | -0.1517 | 0.4595 | 0.3725 | 0.0426* |
| Middle frontal gyrus | 0.1820 | 0.3356 | -0.0509 | 0.7892 | 0.2299 | 0.2216 | 0.6519 | < 0.001** | 0.2873 | 0.1308 | 0.1914 | 0.3110 | -0.0445 | 0.8152 | -0.0910 | 0.6583 | 0.3225 | 0.0822 |
| Inferior frontal gyrus | 0.1830 | 0.3920 | -0.1047 | 0.6262 | 0.3316 | 0.1134 | 0.6813 | < 0.001** | 0.3439 | 0.1081 | 0.0242 | 0.9106 | -0.0815 | 0.7051 | -0.2712 | 0.2107 | 0.2601 | 0.2196 |
| Orbital gyrus | 0.0775 | 0.7126 | -0.1286 | 0.5402 | 0.1290 | 0.5388 | 0.5584 | 0.0037** | 0.0782 | 0.7164 | 0.1690 | 0.4193 | -0.0029 | 0.9889 | -0.2162 | 0.3103 | 0.3742 | 0.0654 |
| Gyrus rectus | 0.0723 | 0.7313 | -0.1664 | 0.4268 | 0.1968 | 0.3458 | 0.6452 | < 0.001** | 0.1536 | 0.4737 | 0.0950 | 0.6514 | -0.0653 | 0.7563 | -0.1337 | 0.5335 | 0.4165 | 0.0384* |
| Caudate nucleus | 0.1604 | 0.3973 | 0.0420 | 0.8256 | -0.0152 | 0.9364 | 0.4846 | 0.0066** | 0.4281 | 0.0205* | -0.0420 | 0.8258 | 0.1270 | 0.5037 | -0.3881 | 0.0501 | 0.4519 | 0.0122* |
| Putamen | 0.0322 | 0.8660 | -0.0787 | 0.6792 | -0.1541 | 0.4162 | 0.4969 | 0.0052** | 0.5608 | 0.0016** | 0.0454 | 0.8117 | 0.2364 | 0.2085 | -0.2825 | 0.1621 | 0.3320 | 0.0731 |
| Globus pallidus | 0.0752 | 0.6931 | -0.1114 | 0.5579 | -0.2180 | 0.2472 | 0.4851 | 0.0066** | 0.4286 | 0.0204* | 0.1433 | 0.4500 | 0.1390 | 0.4637 | -0.1855 | 0.3642 | 0.4075 | 0.0254* |
| Thalamus | 0.0964 | 0.6125 | -0.1223 | 0.5196 | -0.0387 | 0.8391 | 0.5080 | 0.0042** | 0.3836 | 0.0399* | 0.0560 | 0.7689 | -0.0837 | 0.6601 | -0.1364 | 0.5065 | 0.1928 | 0.3075 |
| Substantia nigra | -0.0603 | 0.7518 | -0.0400 | 0.8337 | 0.0105 | 0.9561 | 0.3889 | 0.0337* | 0.3523 | 0.0609 | -0.0878 | 0.6445 | 0.2831 | 0.1295 | -0.3714 | 0.0618 | 0.2197 | 0.2433 |

Correlations between clinical and pathological variables of 30 pAGD cases were assessed with Spearman's rank-order correlation test. Because all diffuse form pAGD cases fit the criteria of Saito AG stage III, the diffuse form was regarded as Saito AG stage III in statistical analyses. NFT: neurofibrillary tangle, AG: argyrophilic grain, LATE-NC: limbic-predominant age-related TDP-43 encephalopathy pathologic change, NL: neuronal loss. *: $p < 0.05$, **: $p < 0.01$.

**Supplementary table 4. Additional multivariate analyses of predictors for neuronal loss in the amygdala, frontal cortex, striatum, and substantia nigra in pAGD and PART cases**

| **Multivariate ordered logistic regression analyses** | | | |
|---|---|---|---|
| | Odds ratio | 95% CI | p value |
| Amygdala (n = 63) [a)] | | | |
| Age at death | 1.0180 | 0.9792-1.0584 | 0.3673 |
| Braak NFT stage | 0.9438 | 0.4634-1.9223 | 0.8734 |
| Saito AG stage | 5.2730 | 2.2509-12.3526 | **< 0.001**** |
| GFA stage in the amygdala | 0.0499 | 0.0015-1.6130 | 0.0909 |
| LATE-NC stage | 0.9936 | 0.4076-2.4225 | 0.9888 |
| | | | |
| Middle frontal gyrus (n = 63) [a)] | | | |
| Age at death | 0.9688 | 0.8756-1.0719 | 0.5390 |
| Braak NFT stage | 0.4938 | 0.1863-1.3088 | 0.1559 |
| Saito AG stage | 4.2678 | 1.5875-11.4737 | **0.0040**** |
| GFA stage in the middle frontal gyrus | 4.9444 | 0.0323-756.3003 | 0.5335 |
| LATE-NC stage | 1.2979 | 0.4458-3.7790 | 0.6325 |
| | | | |
| **Binomial logistic regression analysis** | | | |
| Caudate nucleus (n = 63) [b)] | | | |
| Age at death | 0.8134 | 0.6585-1.0049 | 0.0555 |
| Braak NFT stages III-IV | 10.0945 | 0.1704-597.8869 | 0.2669 |
| Saito AG stage III [c)] | 114.5962 | 1.4586-9003.1459 | **0.0332*** |
| Presence of GFAs in the caudate nucleus [d)] | 0.0000 | 0.0000-15314.8940 | 0.0941 |
| LATE-NC stage 2 [e)] | 738.6657 | 2.3224-2.3494.E+05 | **0.0247*** |
| | | | |
| Putamen (n = 63) [b)] | | | |
| Age at death | 0.8423 | 0.6805-1.0426 | 0.1148 |
| Braak NFT stages III-IV | 0.0966 | 0.0005-18.8232 | 0.3850 |
| Saito AG stage III [c)] | 369.9374 | 2.2451-60957.1736 | **0.0232*** |
| Presence of GFAs in the putamen [d)] | 111.2875 | 0.0000-3.1574.E+28 | 0.8795 |
| LATE-NC stage 2 [e)] | 41.1989 | 0.0403-42125.7008 | 0.2930 |

30 pAGD and 34 PART cases were examined. The GFA status was additionally submitted as an independent variable in each model. CI: confidence interval, NFT: neurofibrillary tangle, AG: argyrophilic grains, LATE-NC: limbic-predominant age-related TDP-43 encephalopathy neuropathologic change (LATE-NC). (a) The dependent variable was a four-point staging system of neuronal loss (none, mild, moderate, and severe. The definitions are noted in the text) on each region. The age at death, Braak NFT stage, Saito AG stage, GFA stage, and LATE-NC stage were submitted as independent variables. (b) The dependent variable was the presence or absence of neuronal loss in each region. c) Because all diffuse-form pAGD cases fit the criteria of Saito AG stage III, the diffuse form was regarded as Saito AG stage III in statistical analyses. d) The presence of GFAs in each region. e) No pAGD or PART case had LATE-NC in stage 3. *: $p < 0.05$, **: $p < 0.01$.

**Supplementary table 5. Univariate binomial logistic regression analyses of predictors for dementia in pAGD and PART cases**

| | OR | 95% CI | *p* |
|---|---|---|---|
| Age at death | 1.12 | 1.04-1.20 | **0.0032**** |
| Braak NFT stages III-IV | 5.71 | 1.50-21.84 | **0.0108*** |
| Thal phase 1 or over | 1.05 | 0.31-3.59 | 0.9410 |
| Saito AG stage I | 2.25 | 0.51-9.94 | 0.2829 |
| Saito AG stage II | 2.25 | 0.51-9.94 | 0.2829 |
| Saito AG stage II or over | 9.30 | 2.29-37.70 | **0.0018**** |
| Density of AGs in the amygdala (per x400 visual field) | | | |
| One to 49 AGs | 2.18 | 0.49-9.63 | 0.3032 |
| 50 to 99 AGs | 12.36 | 1.25-122.62 | **0.0317*** |
| 100 or more AGs | 8.25 | 1.38-49.21 | **0.0206*** |
| Presence of AGs in the amygdala | 21.94 | 4.07-118.28 | **<0.001**** |
| Density of AGs in the CA1 (per x400 visual field) | | | |
| One to 49 AGs | 2.00 | 0.39-10.28 | 0.4067 |
| 50 to 99 AGs | 2.00 | 0.39-10.28 | 0.4067 |
| 100 or more AGs | 2.46 | 1.37-4.41 | **0.0025**** |
| Presence of AGs in the CA1 | 16.90 | 3.22-88.68 | **<0.001**** |
| Presence of AGs in the lateral occipitotemporal gyrus | 13.95 | 3.08-63.13 | **<0.001**** |
| Presence of AGs in the inferior temporal gyrus | 19.83 | 3.29-119.41 | **0.0011**** |
| Presence of AGs in the insular cortex | 17.60 | 3.46-89.51 | **<0.001**** |
| Presence of LATE-NC | 8.50 | 1.43-50.66 | **0.0188*** |
| LATE-NC stage 2[a)] | 12.73 | 1.28-126.14 | **0.0297*** |
| Neuronal loss stages 2-3 in the amygdala | 34.02 | 3.60-321.60 | **0.0021**** |

51 cases (23 pAGD cases and 28 PART cases) that lacked two or more lacunae or larger infarctions in the cortex and/or subcortical regions were included in univariate analyses. Because all diffuse form pAGD cases fit the criteria of Saito AG stage III, the diffuse form was regarded as Saito AG stage III in statistical analyses. OR: odds ratio, CI: confidence interval, NFT: neurofibrillary tangle, AG: argyrophilic grains, LATE-NC: limbic-predominant age-related TDP-43 encephalopathy neuropathologic change. a) No pAGD or PART case had LATE-NC in stage 3. *: $p < 0.05$, **: $p < 0.01$.

**Supplementary table 6. Eight previously reported pAGD cases having AGs in the frontal cortex and subcortical nuclei**

| | | 1 | 2 | 3 | 4 | 5 | 6 | 7 | 8 |
|---|---|---|---|---|---|---|---|---|---|
| References | | Ishihara et al. [20] | Inoue et al. [19] | Hokelekli et al. [16] | Itagaki et al. [21] | Muarage et al. (case 1) [35] | Muarage et al. (case 2) [35] | Tsuchiya et al. [62] | Arakawa A et al. (case 1) [4] |
| Sex | | M | M | M | F | F | F | F | M |
| Age at onset (y) | | 49 | 52 | 55 | 61 | 62 | 72 | 74 | 81 |
| Age at death (y) | | 54 | 55 | 82 | 68 | 76 | 79 | 89 | 84 |
| Disease duration (y) | | 5 | 3 | 27 | 7 | 6 | 7 | 15 | 3 |
| Initial symptoms | | character change, behavioral change | quadri-paresis, bulbar palsy, cognitive decline | personality change | memory loss | obsession, changes of eating habits, apathy | memory impairment | memory impairment | forgetfulnes s |
| Parkinsonism | | - | + | + | n.d. | - | + | - | + |
| Braak NFT stage | | I | I | III | n.d. (rare) | n.d. | 0 | II | II |
| Aβ deposits | | n.d. | - | Thal phase 0 | - | neocortical, focal | occipital, focal | - | Thal phase 4 |
| Lewy bodies | | +[a] | - | | n.d. | +[b] | - | n.d. | - |
| TDP-43 | | n.d. | - | - | n.d. | n.d. | n.d. | n.d. | LATE-NC stage 2 |
| AGs | Frontal cortex | + | + | + | + | + | + | + | n.d. |
| | Primary motor cortex | n.d. | + | n.d. | + | + | + | n.d. | n.d. |
| | Basal ganglia | n.d. | - | + | + | + | + | + | + |
| | Brain stem | + | + | + | + | - | - | n.d. | + |
| Neu-ronal loss | CA1 | + (severe) | - | n.d. | - | n.d. | n.d. | + (prominent) | n.d. |
| | Substantia nigra | + (severe) | + (moderate) | n.d. | + | - | n.d. | + (obvious) | + |

y: years, NFT: neurofibrillary changes. -: absent, +: present, n.d.: not described. a) Lewy bodies in the substantia nigra and locus coeruleus. b) Rare Lewy bodies in the substantia nigra and dorsal vagal nucleus.
